# Investigation of Potential Mechanisms of Yerba Santa Compounds Against Alzheimer’s Disease

**DOI:** 10.3390/molecules31203757

**Published:** 2026-10-19

**Authors:** Magally Arevalo Navarro, Charles Alfano, Jonathan Cross, Tianhua Zhai, Li Shen, Zuyi Huang

**Affiliations:** 1 Department of Chemical and Biological Engineering, Villanova University, Villanova, PA 19085, USA; 2 Department of Biostatistics, Epidemiology and Informatics, University of Pennsylvania, Philadelphia, PA 19104, USA

**Keywords:** Alzheimer’s disease, Yerba Santa, *Eriodictyon*, molecular docking, statistical validation, neuroinflammation, flavonoids, complement system, natural products

## Abstract

Alzheimer’s disease (AD) is a progressive neurodegenerative disorder in which chronic neuroinflammation plays a central role in disease initiation and progression. Yerba Santa (*Eriodictyon* spp.) is a medicinal plant with reported anti-inflammatory and neuroprotective properties, although its molecular mechanisms remain poorly understood. This study investigated whether chemically characterized Yerba Santa compounds show predicted docking interactions with neuroinflammation-related protein targets implicated in AD. Thirty-six ligand structures, representing 30 mass spectrometry peak assignments, were docked against 30 neuroinflammation-related proteins using the MolSoft ICM molecular docking platform. To improve reproducibility, each ligand–target pair was evaluated by replicated docking simulations followed by statistical analysis, with apo and holo targets evaluated against separate statistical references and expanded docking where applicable. Under the apo −25 kcal/mol screening reference, 11 ligand–target pairs were retained after BH-FDR correction and expanded docking for the apo C1r-C1s (PDB:6F39). Separately, 23 holo ligand–target pairs were retained after comparison with their corresponding redocked native-ligand references: Complement C1q (PDB:2JG8), GABA-A receptor α1β2γ2 (PDB:6D6U), and NLRP3 (PDB:7PZC). The pairs retained under these separate criteria involved multiple Yerba Santa compounds, predominantly flavonoids, while Rosmarinic acid (L21) was retained for both Complement C1q (PDB:2JG8) and NLRP3 (PDB:7PZC). These findings support a selective predicted multi-target interaction profile for Yerba Santa compounds and identify complement signaling, GABA-A receptor-associated neurotransmission, and NLRP3 inflammasome signaling as testable pathways for future experimental studies of neuroinflammation in AD.

## 1. Introduction

In recent decades, medical advances have substantially reduced mortality from cancer, cardiovascular diseases, diabetes, and infectious diseases such as HIV, for which mortality has declined by nearly 66%. In contrast, mortality attributable to Alzheimer’s disease (AD) has increased sharply in aging populations, rising by more than 134% between 2000 and 2024 [1]. AD currently affects approximately 5.2% of individuals aged 65 to 74, 13.8% of those aged 75 to 84, and 35.8% of individuals over 85 years of age, and it ranks as the fifth leading cause of death among Americans aged 65 and older and the sixth leading cause of death overall in the US [1]. These trends underscore AD as one of the most urgent unmet medical challenges associated with aging. AD is a progressive neurodegenerative disorder characterized by memory loss and impairment of higher cognitive functions. For more than three decades, the accumulation of β-amyloid (Aβ) plaques and neurofibrillary tangles has dominated diagnostic frameworks and therapeutic strategies [2]. However, growing evidence suggests that these pathological hallmarks may not represent the earliest or sole drivers of disease. The updated Amyloid Cascade Hypothesis 2.0 proposes a more complex, multifactorial disease trajectory involving additional molecular and cellular processes [3,4], and recent studies indicate that broader protein networks contribute to disease initiation and progression [5]. These observations highlight the need to explore alternative and complementary therapeutic pathways.

Neuroinflammation has emerged as a central contributor to AD pathogenesis. Microglia and astrocytes, which initially exert protective functions, can transition to a chronically activated and dysregulated pro-inflammatory state that accelerates neuronal damage. This inflammatory milieu is tightly linked to mitochondrial dysfunction, which impairs neuronal energy production, increases oxidative stress, and promotes synaptic loss and neuronal apoptosis. These processes form self-reinforcing feedback loops that exacerbate amyloid and tau pathology, accelerating disease progression. Recent genomic and transcriptomic analyses have identified inflammation-related protein targets relevant to Alzheimer’s disease, including complement cascade components (C3, C4b, C5, C9), immune and neurotransmitter receptors such as DRD2 and C5aR1, and GABA-A receptor subunits [6]. Together, these findings establish neuroinflammatory pathways as biologically relevant targets for investigating potential therapeutic approaches in AD. These observations provide a rationale for investigating natural compounds with reported anti-neuroinflammatory and neuroprotective activities as potential interactors with inflammation-related protein targets implicated in AD.

Yerba Santa (*Eriodictyon californicum*) contains structurally diverse flavanones, flavones, flavonols, prenylated benzoic acids, and polyphenolic acids [7], many of which have antioxidant and anti-inflammatory properties relevant to inflammation-driven aging. These reported biological activities provide a rationale for investigating Yerba Santa compounds in the context of inflammation-related pathways implicated in AD. Yerba Santa was selected for this study because its compounds have been well isolated, characterized, and consistently reported, providing a defined and reproducible chemical space for systematic ligand–target analysis. Despite growing interest in its bioactive compounds, the molecular mechanisms by which Yerba Santa compounds may interact with inflammation-related AD targets remain poorly defined. In particular, their predicted interactions with specific neuroinflammation-related protein targets implicated in AD have not been systematically evaluated. In this context, this study investigates the California native medicinal plant *Eriodictyon californicum* (Yerba Santa) as a source of bioactive compounds with the potential to interact with targets involved in neuroinflammatory pathways relevant to AD [8].

Ligand–protein molecular docking is a commonly employed computational approach to investigate potential interactions between compounds (e.g., Yerba Santa compounds) and proteins (e.g., proteins involved in neuroinflammation). Indeed, molecular docking has been applied to investigate interactions between small molecules and protein targets implicated in human diseases [9]. By modeling ligand binding within structurally defined protein pockets, docking enables hypothesis-driven prioritization of Yerba Santa compounds based on predicted interaction geometry, energetic favorability, and target specificity. However, because docking algorithms rely on stochastic sampling and empirical scoring functions [10], individual docking scores can be sensitive to initial conditions and local energy minima. As a result, single-run docking outcomes may overestimate isolated interactions or fail to distinguish reproducible binding trends from stochastic artifacts. Integrating replicated docking simulations with statistical evaluation [11,12] addresses this limitation by identifying predicted ligand–target interactions with docking score patterns that are consistent across independent docking analyses and increased sampling levels, thereby enhancing confidence in their reproducibility. Such replicated docking frameworks are particularly well suited for evaluating medicinal plants, where structurally diverse phytochemicals may exhibit predicted interactions with overlapping or complementary protein networks. In the context of Alzheimer’s disease, which may arise from the convergence of neuroinflammatory, oxidative, and metabolic pathways, this approach provides a rational and scalable means to link phytochemical diversity to target-specific biological hypotheses and to prioritize compounds for further investigation. Accordingly, a Monte Carlo-based flexible docking strategy was employed to capture the conformational flexibility of both ligands and binding sites, enabling hypothesis-driven analysis of predicted multi-target interactions. To reduce stochastic sampling artifacts and improve reproducibility, ligand–target pairs meeting the predefined screening and statistical criteria during the initial screening stage were further evaluated using replicate docking analyses and increased sampling levels.

An additional challenge in interpreting molecular docking results is the use of a universal docking score threshold across structurally diverse protein targets. Docking scores depend on protein structure, binding-pocket geometry, ligand properties, and scoring function assumptions. Consequently, a single numerical cutoff may not provide an equally appropriate reference for every protein target. The availability of experimentally characterized ligand-bound protein structures provides an opportunity to establish target-specific computational references through native-ligand redocking. In contrast, apo structures without co-crystallized ligands lack an equivalent structural reference and require a different screening strategy. Distinguishing between these two structural categories may therefore improve the interpretability of docking score comparisons while reducing reliance on an arbitrary universal cutoff. Accordingly, we developed a structure-dependent docking and statistical evaluation framework that distinguishes between apo and holo protein targets. For apo targets, a predefined ICM docking score criterion was used for initial screening, whereas holo targets were evaluated relative to docking score distributions obtained from their corresponding co-crystallized native ligands. Replicated molecular docking, statistical testing, multiple-comparison correction, and expanded sampling where applicable were integrated to prioritize ligand–target interactions exhibiting reproducible predicted docking behavior. By addressing this gap, the work provides a framework for prioritizing predicted ligand–target interactions in complex phytochemical mixtures and a foundation for future experimental validation. Because the apo and holo analyses use different references, their *p*-values and adjusted *p*-values are not directly comparable across the two structural categories.

## 2. Results

### 2.1. Docking Score Distributions and Assessment of Docking Repetition Number (n = 10)

**Figure 1:**
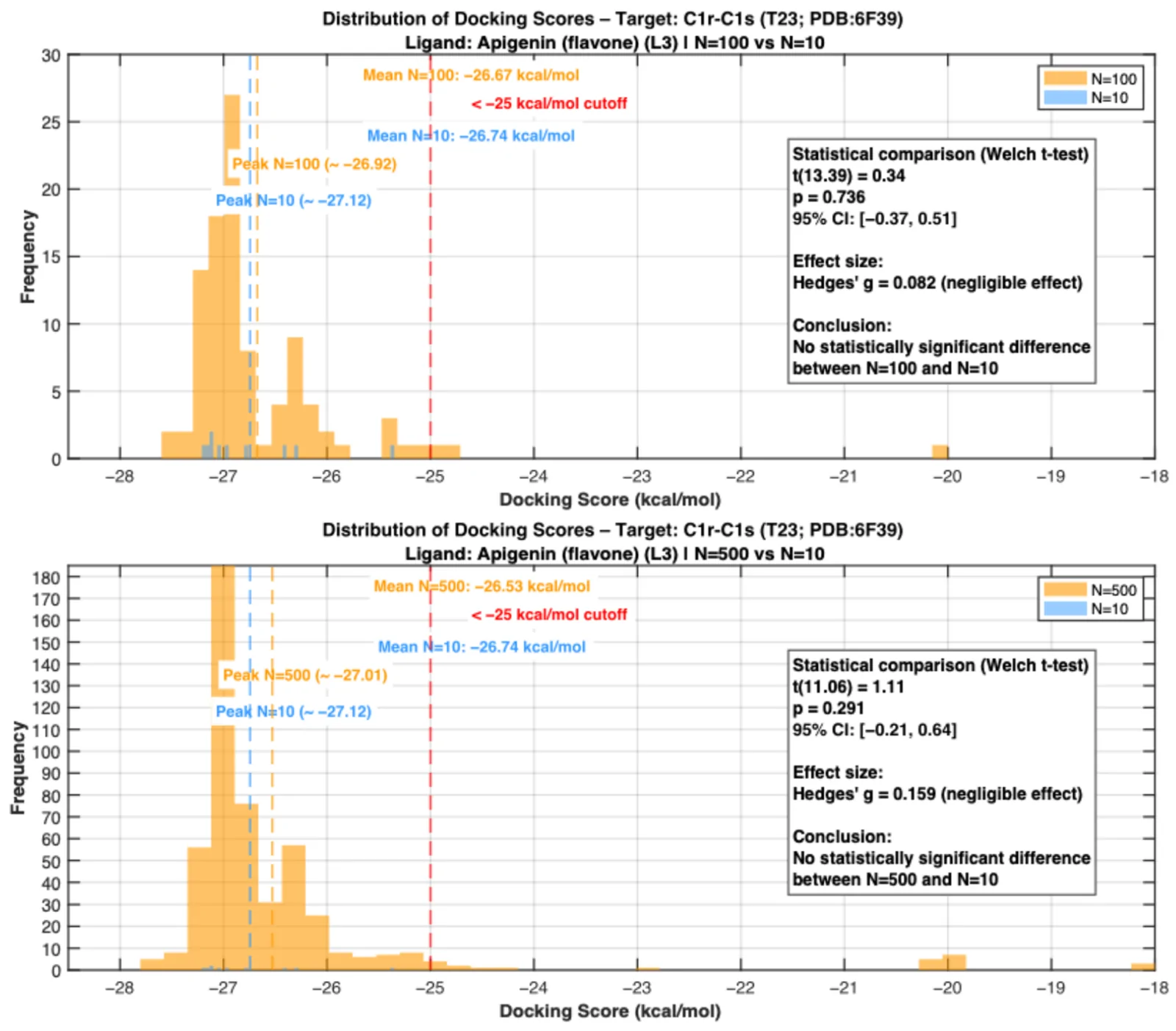
Docking score distributions for ligand Apigenin (L3) against target C1r-C1s (PDB: 6F39; T23) across different sampling depths (*n* = 10 vs. *n* = 100 and *n* = 10 vs. *n* = 500). Comparable score distributions and mean values indicate stability of the docking score distribution with increased sampling, supporting *n* = 10 for the initial docking analysis.

To evaluate the stability of docking score distributions and justify the use of 10 independent dockings per ligand–target pair, expanded docking ensembles were examined for ligand Apigenin (indexed as Ligand-3, i.e., L3) against target C1r-C1s (PDB:6F39; indexed as Target 23, i.e., T23) as a demonstration example. This ligand–target pair was docked using increased sampling densities of 100 and 500 independent dockings, and the resulting score distributions were compared with those obtained using 10 runs. As shown in Figure 1, increasing the number of docking repetitions did not materially alter the overall shape of the distributions or their central tendencies. Two-sample Welch’s *t*-tests revealed no statistically significant differences between the mean docking scores obtained with *n* = 10 and *n* = 100 (*p* = 0.736) or between *n* = 10 and *n* = 500 (*p* = 0.291). The observed mean differences (<0.21 kcal·mol^−1^) were small relative to the inherent variability of the docking scores (SD*_n_*_=10_ = 0.58, SD*_n_*_=100_ = 0.865, and SD*_n_*_=500_ = 1.35 kcal·mol^−1^), indicating that increased sampling depth did not substantially shift the estimated docking score distribution. Together, these results support the adequacy of *n* = 10 independent docking runs for the initial docking analysis.

A total of 36 Yerba Santa ligands (corresponding to 30 MS peak assignments) were docked against 30 neuroinflammation-related protein targets, generating 1080 unique ligand–target combinations and 10,800 docked poses across 10 independent runs per ligand. All 10 independent docking scores for each ligand–target pair were retained and reported, as illustrated for the apo target C1r-C1s (PDB:6F39; T23) and the holo target Arf6 (PDB:1E0S, T1) in Table 1 as representative examples. Docking score distributions were then examined to characterize the overall docking score landscape for individual targets across all screened ligands. The resulting distributions varied among targets in their central tendencies, spread, and distributional shape. Because a universal numerical cutoff may not be equally appropriate for structurally different protein targets, the 30 protein structures were separated into 21 apo and nine holo targets according to the availability of a co-crystallized native ligand. The predefined ICM docking score criterion of −25 kcal/mol was retained for the initial screening of apo targets only. For holo targets, the corresponding native ligands were redocked to generate target-specific computational reference distributions, which were subsequently used for statistical comparison with the Yerba Santa ligand docking scores. Accordingly, the −25 kcal/mol criterion was not applied to the holo target analysis. The two structural categories were evaluated separately using the statistical procedures described in Section 2.2 and Section 2.3.

**Figure 2:**
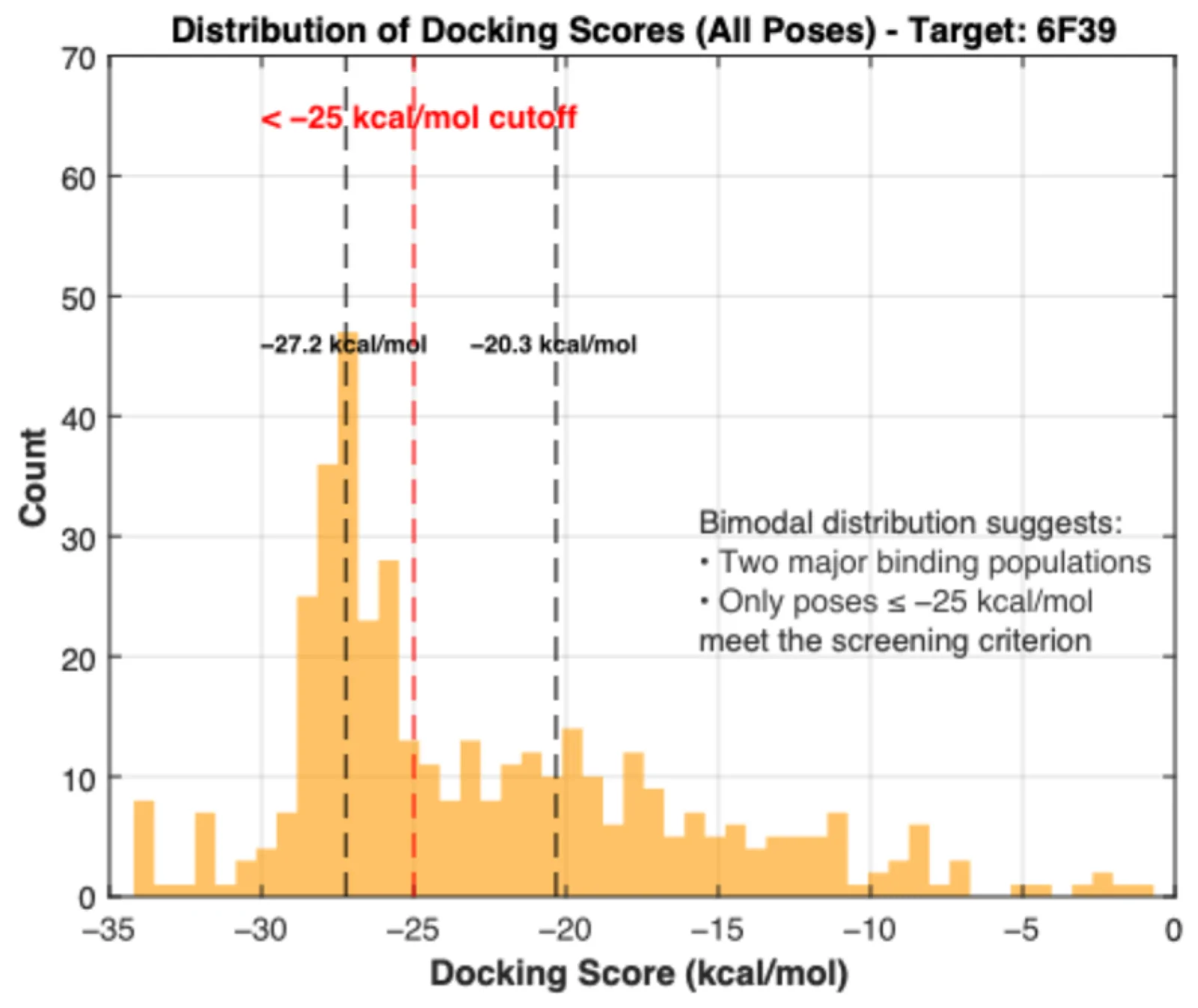
The distribution of docking scores for target C1r-C1s (PDB:6F39; T23). A histogram of docking scores obtained from all poses generated across ten independent docking runs for 36 Yerba Santa ligands. The dashed red line indicates the predefined −25 kcal·mol^−1^ ICM screening criterion used for ligand–target prioritization. Gaussian mixture modeling supported a two-component distribution, with component means at approximately −27.2 and −20.3 kcal·mol^−1^.

Because these distributions aggregate all ligands tested for each target, they represent the overall scoring landscape of each protein across the screened ligand set. For target C1r-C1s (PDB:6F39; T23), a histogram constructed from docking scores obtained for all 36 Yerba Santa ligands showed a bimodal distribution (Figure 2). To quantitatively assess this behavior, Gaussian mixture modeling was applied, and one- and two-component models were compared using the Bayesian Information Criterion (BIC), which supported the two-component model. The two components were centered at approximately −27.2 and −20.3 kcal·mol^−1^, supporting a two-component representation of the observed docking score distribution. Histograms of this type were obtained for individual protein targets to characterize their docking score distributions and identify ligand–target pairs meeting the predefined ICM screening criterion of ≤−25 kcal/mol.

### 2.2. Statistical Analysis of Apo Target Docking Scores

**Figure 3:**
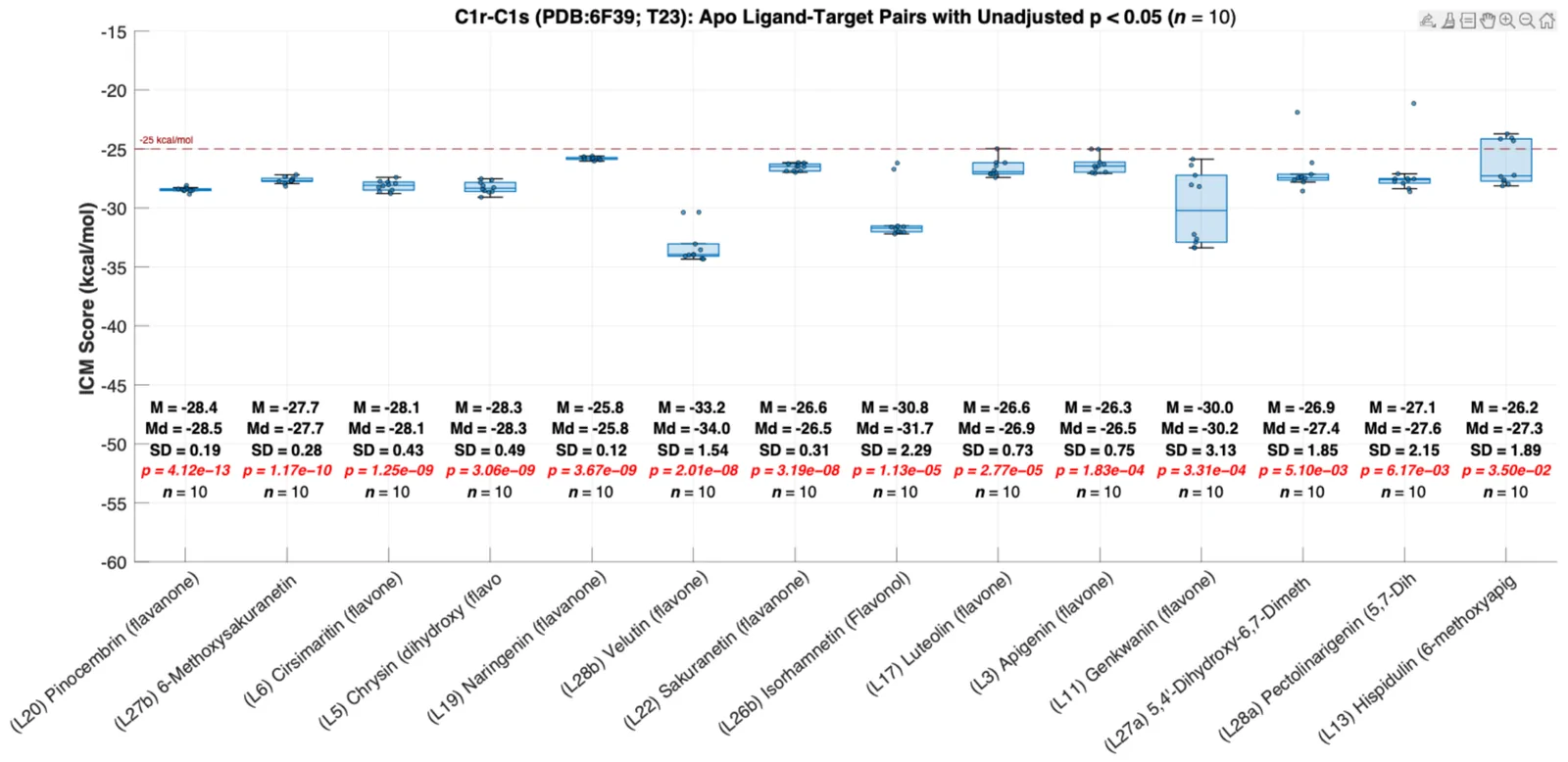
Docking score distributions for the 14 apo ligand–target pairs with an unadjusted *p* < 0.05 in the initial *n* = 10 analysis. Statistical significance was evaluated using a one-sample, left-tailed Student’s *t*-test against the statistical reference value of −25 kcal mol^−1^. The dashed red line indicates the −25 kcal mol^−1^ statistical reference value. M, Md, SD, *n*, and the corresponding unadjusted *p*-value are shown for each ligand–target pair.

**Figure 4:**
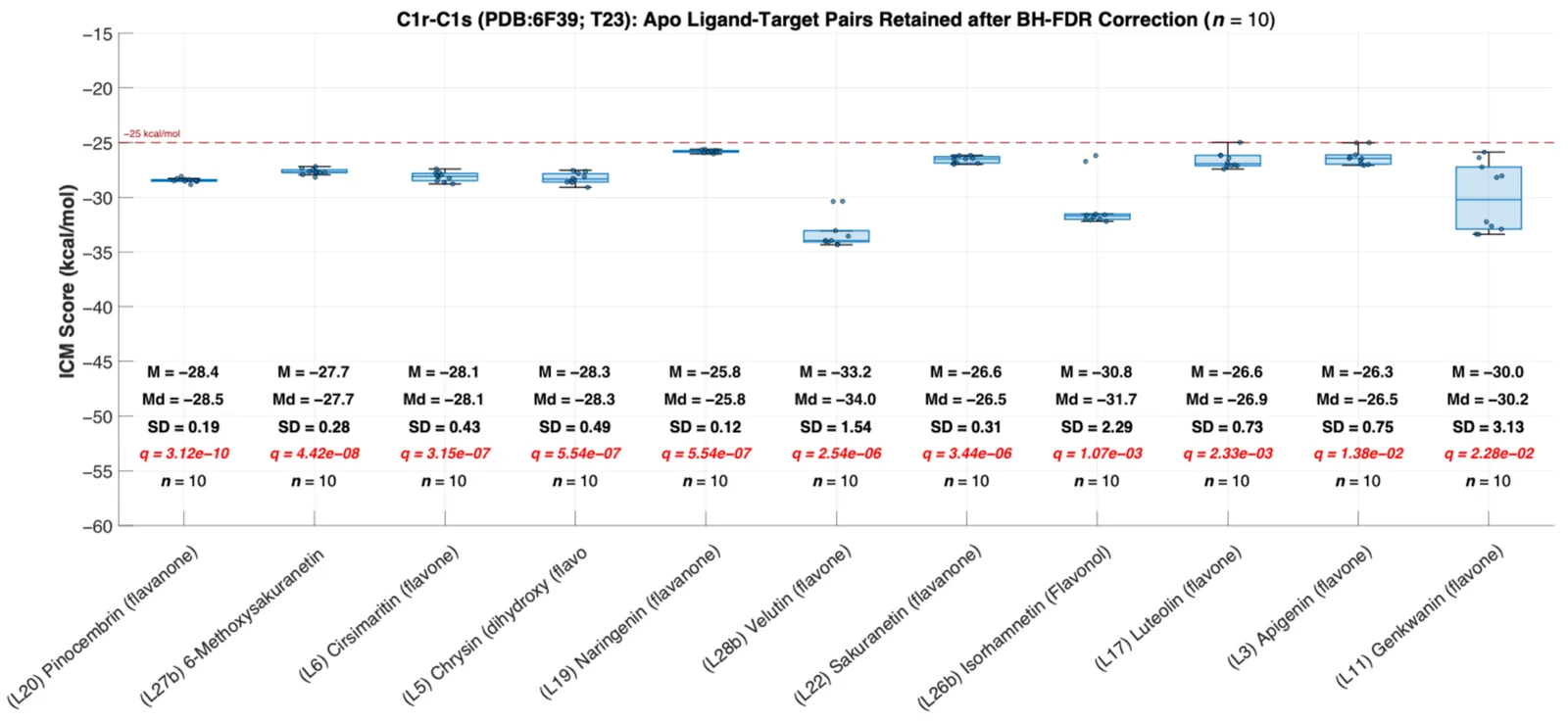
Docking score distributions for the 11 apo ligand–target pairs remaining statistically significant after Benjamini–Hochberg false discovery rate (BH-FDR) correction across the 756 initial *n* = 10 apo comparisons. All 11 interactions involved C1r-C1s (PDB:6F39; T23). The dashed red line indicates the −25 kcal mol^−1^ statistical reference value. M, Md, SD, *n*, and the corresponding BH-FDR-adjusted q-value are shown for each ligand–target pair. Statistical significance after multiple-testing correction was defined as q < 0.05.

**Figure 5:**
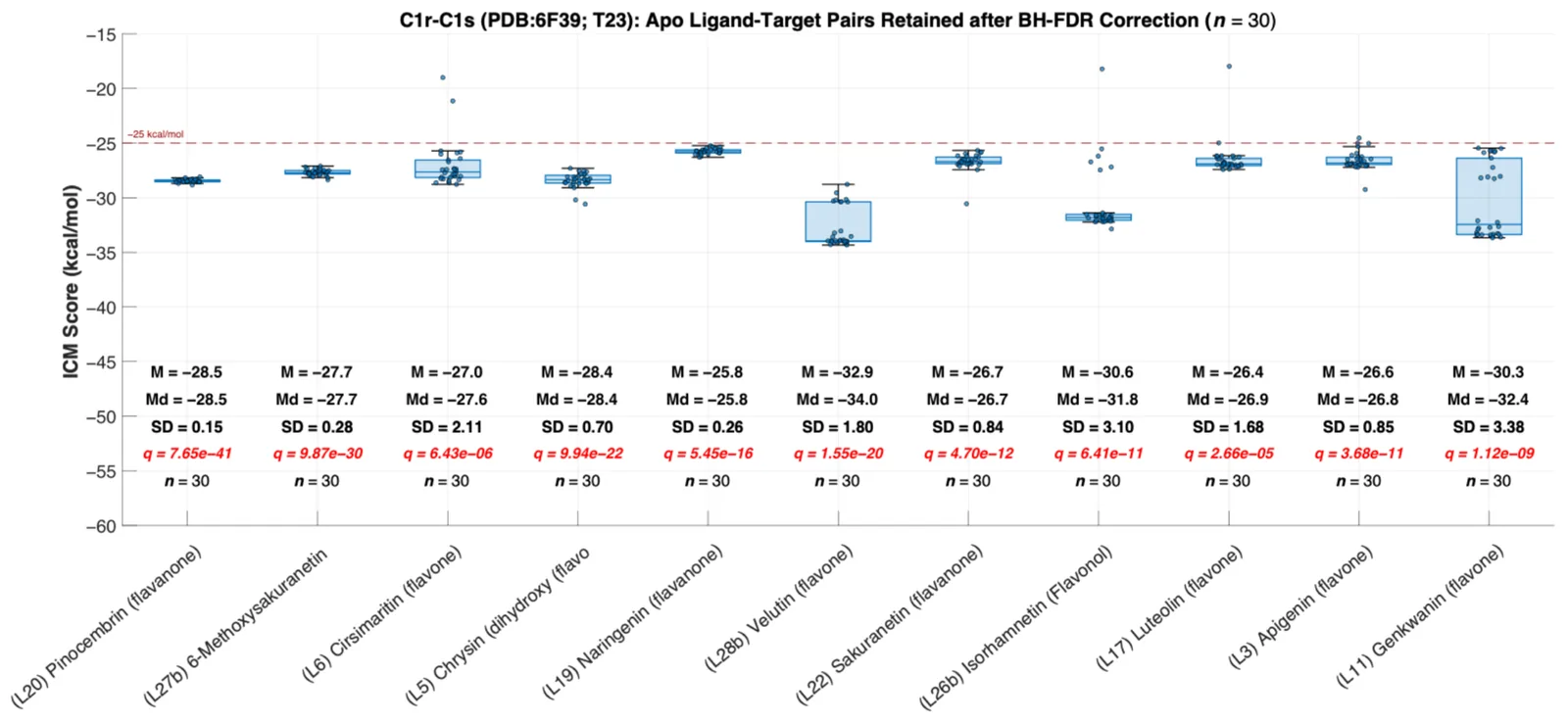
Expanded *n* = 30 docking score analysis of the 11 C1r-C1s (PDB:6F39; T23) ligand–target pairs retained after BH-FDR correction in the initial *n* = 10 analysis. The *n* = 30 results were evaluated using a one-sample, left-tailed Student’s *t*-test against −25 kcal mol^−1^, followed by separate BH-FDR correction across the 11 follow-up comparisons. The dashed red line indicates the −25 kcal mol^−1^ statistical reference value. M, Md, SD, *n*, and the corresponding BH-FDR-adjusted q-value are shown for each ligand–target pair. All 11 pairs remained statistically significant at q < 0.05.

**Figure 6:**
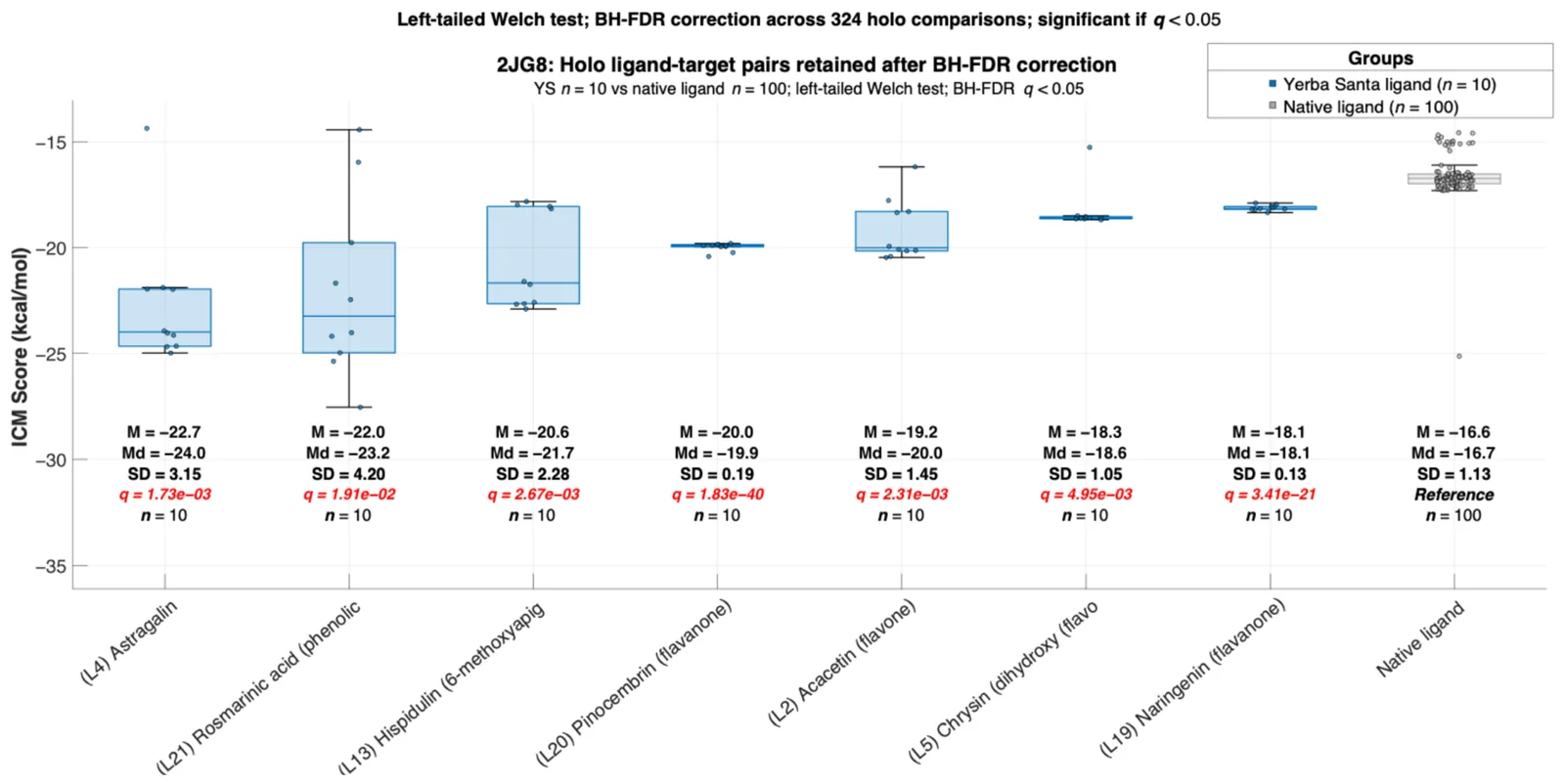
Holo ligand–target docking score comparisons for Complement C1q (PDB:2JG8) retained after BH-FDR correction. Yerba Santa ligand docking scores (*n* = 10) were compared with the native-ligand redocking scores (*n* = 100) using left-tailed Welch two-sample *t*-tests, followed by BH-FDR correction across all 324 holo comparisons. Seven ligand–target pairs remained statistically significant (q < 0.05). M, Md, SD, *n*, and BH-FDR-adjusted q-values are shown.

**Figure 7:**
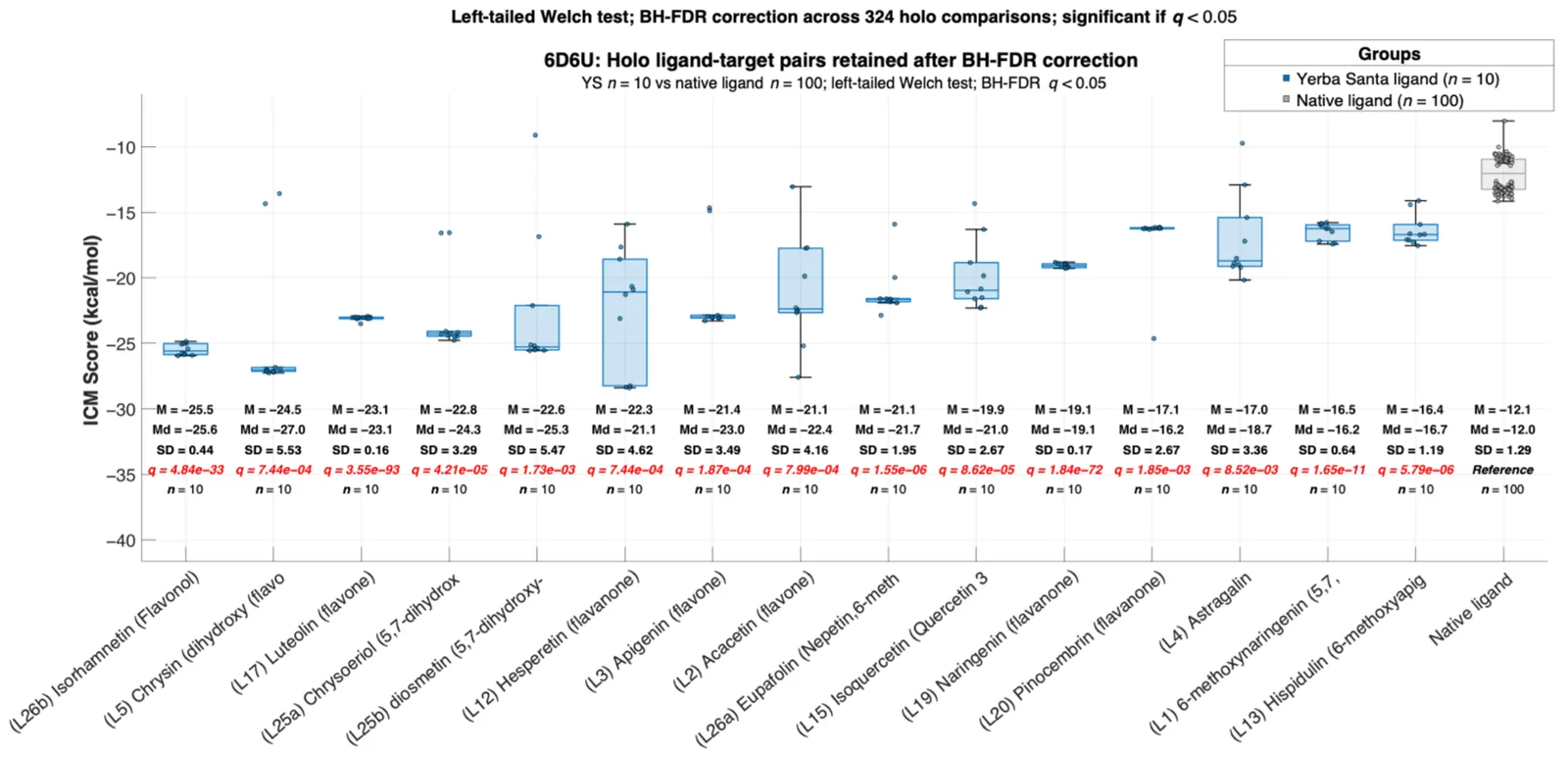
Holo ligand–target docking score comparisons for GABA-A receptor α1β2γ2 (PDB:6D6U) retained after BH-FDR correction. Yerba Santa ligand docking scores (*n* = 10) were compared with the native-ligand redocking scores (*n* = 100) using left-tailed Welch two-sample *t*-tests, followed by BH-FDR correction across all 324 holo comparisons. Fifteen ligand–target pairs remained statistically significant (q < 0.05). M, Md, SD, *n*, and BH-FDR-adjusted q-values are shown.

**Figure 8:**
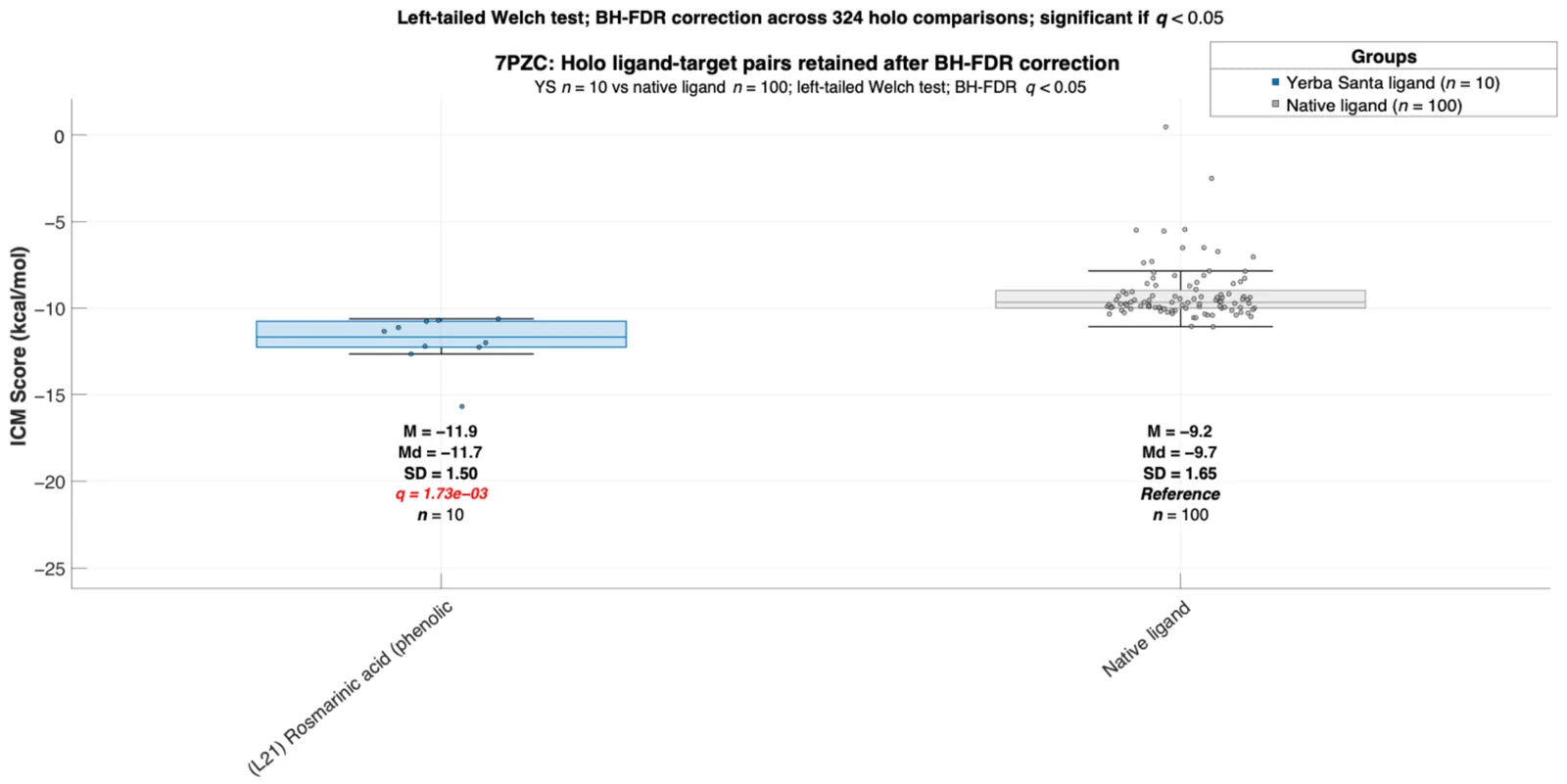
Holo ligand–target docking score comparison for NLRP3 (PDB:7PZC) retained after BH-FDR correction. Yerba Santa ligand docking scores (*n* = 10) were compared with the native-ligand redocking scores (*n* = 100) using a left-tailed Welch two-sample *t*-test, followed by BH-FDR correction across all 324 holo comparisons. One ligand–target pair remained statistically significant (q < 0.05). M, Md, SD, *n*, and the BH-FDR-adjusted q-value are shown.

For the 21 apo targets, statistical analysis was performed on the initial *n* = 10 docking results, comprising 756 ligand–target comparisons. A one-sample, left-tailed Student’s *t*-test was used to determine whether the mean docking score for each ligand–target pair was significantly below the statistical reference value of −25 kcal mol^−1^. Of the 756 comparisons, 14 showed an unadjusted *p* < 0.05. The docking score distributions and corresponding unadjusted *p*-values for these initially significant ligand–target pairs are shown in Figure 3. To improve readability and avoid overlapping labels, ligand names on the X-axes of Figure 3, Figure 4, Figure 5, Figure 6, Figure 7 and Figure 8 were intentionally shortened. The corresponding full ligand names are provided in Table 6.

To account for multiple comparisons, Benjamini–Hochberg false discovery rate (BH-FDR) correction was applied across all 756 initial apo comparisons. Following BH-FDR correction, 11 ligand–target pairs remained statistically significant (q < 0.05), all involving C1r-C1s (PDB:6F39; T23). The docking score distributions and corresponding BH-FDR-adjusted q-values for these 11 interactions are shown in Figure 4.

To evaluate whether the statistical results persisted with increased sampling, docking was expanded from *n* = 10 to *n* = 30 for the 11 ligand–target pairs retained after initial BH-FDR correction. The *n* = 30 docking scores were re-evaluated using the same one-sample, left-tailed Student’s *t*-test against −25 kcal mol^−1^, followed by a separate BH-FDR correction across the 11 follow-up comparisons. All 11 ligand–target pairs remained statistically significant after BH-FDR correction (q < 0.05). The *n* = 10 and *n* = 30 statistical results for these 11 ligand–target pairs are summarized in Table 2. The expanded docking score distributions and corresponding BH-FDR-adjusted q-values are shown in Figure 5. Thus, the statistical results for all 11 retained C1r-C1s interactions persisted when the number of docking runs increased from 10 to 30.

### 2.3. Native-Ligand Redocking and Statistical Analysis of Holo Targets

For the 9 holo targets, the corresponding crystallographic native ligands were redocked using *n* = 100 independent runs to establish target-specific computational reference distributions. The resulting native-ligand docking scores were used as references for comparison with the Yerba Santa ligand docking scores (*n* = 10) for the same target. Unlike the apo target analysis, the −25 kcal mol^−1^ statistical reference value was not applied to the holo targets. Instead, each holo target was evaluated using its corresponding native-ligand docking score distribution as a target-specific computational reference. Redocking reproduced the crystallographic binding poses for all 9 holo targets, with RMSD values <0.5 Å between the redocked and experimental native-ligand poses. These results support the ability of the docking protocol to recover the experimentally observed binding orientations.

A total of 324 holo ligand–target comparisons were evaluated using a left-tailed Welch two-sample *t*-test to determine whether the mean Yerba Santa ligand docking score was lower than the mean native-ligand docking score for the corresponding target. Following Benjamini–Hochberg false discovery rate (BH-FDR) correction across all 324 comparisons, 23 ligand–target pairs remained statistically significant (q < 0.05). These comprised 7 interactions with Complement C1q (PDB:2JG8), 15 with GABA-A receptor α1β2γ2 (PDB:6D6U), and 1 with NLRP3 (PDB:7PZC), while no ligand–target pairs remained statistically significant for the other six holo targets. The docking score distributions and corresponding BH-FDR-adjusted q-values for the retained interactions are shown in Figure 6, Figure 7 and Figure 8. These comparisons showed more negative mean docking scores for the retained Yerba Santa ligand–target pairs relative to the corresponding native-ligand computational reference and were not interpreted as evidence of experimentally stronger binding.

### 2.4. Structural and Chemical Features of Yerba Santa Ligands

The ligand–target pairs retained under the separate apo and holo criteria are summarized in Table 3. Following BH-FDR correction and expanded docking of the selected apo interactions, 11 apo ligand–target pairs were retained for C1r-C1s (PDB:6F39). For the holo targets, 23 ligand–target pairs remained significant after BH-FDR correction, comprising 7 interactions with Complement C1q (PDB:2JG8), 15 with GABA-A receptor α1β2χ2 (PDB:6D6U), and 1 with NLRP3 (PDB:7PZC).

The Yerba Santa ligands included in the docking analysis were examined to characterize their core structural classes, functional groups, and predicted interaction capabilities. Predicted interaction capabilities were inferred from ligand functional group composition and included the potential for hydrogen bonding, electrostatic, π-π stacking, and hydrophobic interactions.

Table 4 summarizes the structural classification, key chemical features, predicted interaction types, and 2D structures of the Yerba Santa ligands retained in the statistically supported apo and/or holo ligand–target comparisons after BH-FDR correction. Structural and chemical information for the remaining Yerba Santa ligands included in the docking library but not meeting this criterion is provided in the Supplementary Materials.

The retained ligands included flavones, flavanones, methoxylated flavonoids, and other phenolic structures. Their functional groups included hydroxyl, methoxy, carbonyl, and, where present, carboxyl groups, together with aromatic systems that may support hydrogen bonding, electrostatic, π-π stacking, and hydrophobic interactions depending on ligand orientation and the local protein environment. These interaction types represent structure-based predictions from ligand chemical features and were not quantified directly from individual docked poses.

## 3. Discussion

### 3.1. Interpretation of Statistically Supported Ligand–Target Interactions

The statistical analysis substantially narrowed the initial ligand–target screening results to a focused set of interactions. Among the apo targets, the retained interactions were concentrated in C1r-C1s (PDB:6F39), with 11 ligand–target pairs remaining significant after expanded docking at *n* = 30. Among the holo targets, 23 ligand–target pairs remained significant after BH-FDR correction, comprising 7 interactions with Complement C1q (PDB:2JG8), 15 with GABA-A receptor α1β2γ2 (PDB:6D6U), and 1 with NLRP3 (PDB:7PZC). The pairs retained under the separate analyses provide a focused set for target-specific structural and biological follow-up, but their distribution across targets does not establish binding preference. A central methodological refinement in the present study was the replacement of a universal docking score cutoff with a structure-dependent reference strategy. The screened protein structures were classified as apo or holo according to the availability of a co-crystallized native ligand. For the 21 apo targets, the predefined −25 kcal/mol ICM screening criterion was retained as a computational reference for ligand prioritization. For the nine holo targets, the corresponding crystallographic native ligands were redocked to generate target-specific docking score distributions, allowing for comparisons with Yerba Santa ligands under the same docking protocol. This distinction reduced reliance on a single numerical cutoff across structurally diverse proteins and incorporated native-ligand computational references where available. Nevertheless, the reference criteria serve different purposes: the apo criterion provides a practical screening benchmark, whereas the holo comparisons characterize docking score behavior relative to a structurally characterized native ligand. Neither approach independently establishes experimental binding affinity or biological activity.

A key feature of this study is the implementation of a replicated docking and statistical evaluation approach to interpret docking results in the context of their inherent stochastic variability. Docking algorithms rely on Monte Carlo-based sampling [13], and individual scores can fluctuate across independent runs, making single-score interpretations unreliable [14]. By performing replicated docking simulations and applying statistical approaches appropriate to apo and holo targets, interactions were evaluated based on their docking score behavior across independent runs rather than individual docking scores. For apo targets, statistically supported interactions were further evaluated using expanded docking from *n* = 10 to *n* = 30, whereas holo interactions were evaluated relative to target-specific native-ligand redocking references. BH-FDR correction was applied to account for multiple comparisons. This distribution-based framework reduces reliance on individual docking scores and provides a reproducible basis for prioritizing predicted ligand–target interactions for further investigation.

Importantly, the objective of this study is not to directly nominate drug candidates, but to establish a mechanistic and hypothesis-generating foundation that connects traditional medicinal use with contemporary target biology. The statistically supported ligand–target interactions identify predicted interactions that remained reproducible within the applied docking and statistical framework and therefore provide a prioritized set for further structural and experimental evaluation. In doing so, this work introduces a scalable strategy for integrating ligand–protein interaction analysis with statistical inference, providing a framework for investigating multi-component natural products against disease-relevant protein targets.

### 3.2. Structural Features of Statistically Supported Yerba Santa Ligands

The statistically supported ligand–target interactions involved compounds representing several structural classes present in Yerba Santa, with flavonoids and flavonoid-related compounds comprising most of the retained ligands. These compounds included flavones, flavanones, flavonols, methoxylated flavonoids, and glycosylated derivatives, together with rosmarinic acid. Flavonoids share a characteristic aromatic scaffold that can support hydrophobic and π-π interactions, while hydroxyl groups provide potential hydrogen-bond donors and acceptors. Methoxy substituents modify the polarity and hydrophobic character of the flavonoid scaffold and may influence its accommodation within different protein environments. These structural characteristics provide plausible chemical features for the predicted interactions observed in the docking analysis, although the contribution of individual interactions was not quantified in this study.

The retained interactions also showed that statistical support was not restricted to a single flavonoid subclass or substitution pattern. Apigenin (L3), Chrysin (L5), Luteolin (L17), Naringenin (L19), Pinocembrin (L20), and Isorhamnetin (L26b) were retained in both the apo and holo analyses, whereas other ligands were supported only for apo or holo targets. This pattern suggests that structurally related Yerba Santa compounds can exhibit different docking score behavior depending on the protein target and binding environment. Therefore, the presence of particular functional groups alone does not account for statistical support across targets, and target-specific structural complementarity remains an important consideration.

Rosmarinic acid (L21), the only non-flavonoid compound among the statistically supported holo interactions, was retained for both Complement C1q (PDB:2JG8) and NLRP3 (PDB:7PZC). Its multiple hydroxyl groups and carboxylic acid functionality provide several potential sites for hydrogen bonding and electrostatic interactions, while its aromatic groups may contribute to hydrophobic and π interactions. However, the present docking analysis does not establish which of these interactions are responsible for the observed statistical support. The retention of rosmarinic acid for two distinct holo targets further illustrates that statistically supported docking behavior was not limited to the flavonoid scaffold.

### 3.3. Biological Relevance of Prioritized Protein Targets

The statistical analyses prioritized four protein targets: C1r-C1s (PDB:6F39) in the apo analysis and Complement C1q (PDB:2JG8), GABA-A receptor α1β2γ2 (PDB:6D6U), and NLRP3 (PDB:7PZC) in the holo analysis. These proteins represent distinct biological pathways relevant to neuroinflammation and Alzheimer’s disease, including complement signaling, neurotransmission, and inflammasome-mediated inflammatory responses [15,16]. The concentration of statistically supported interactions within these targets does not establish functional modulation; rather, it identifies specific protein–ligand systems for which the docking results provide a rationale for further mechanistic and experimental investigation.

The complement-related targets C1r-C1s (PDB:6F39) and Complement C1q (PDB:2JG8) are particularly relevant to the inflammatory component of Alzheimer’s disease because they participate in the classical complement pathway [17,18]. C1q functions as the recognition component of the C1 complex, whereas C1r and C1s participate in downstream complement activation [18]. Importantly, the C1r-C1s structure used in the apo analysis represents the CUB1-EGF-CUB2 region of C1r rather than its catalytic serine-protease domain. Therefore, the 11 statistically supported interactions observed with this structure should be interpreted as predicted structural compatibility with the modeled region and not as evidence of C1r enzymatic inhibition. Similarly, the seven statistically supported interactions with Complement C1q (PDB:2JG8) identify predicted interactions with this complement-related target but do not establish an effect on complement activity.

The other two prioritized holo targets extend the findings to neurotransmission and inflammasome signaling. GABA-A receptor α1β2γ2 (PDB:6D6U), for which 15 ligand–target pairs remained statistically significant, is a major inhibitory neurotransmitter receptor in the adult brain [15]. NLRP3 (PDB:7PZC), for which Rosmarinic acid (L21) was the single statistically supported Yerba Santa ligand, is an intracellular sensor involved in NLRP3 inflammasome formation and inflammatory signaling [19,20]. Notably, 7PZC contains the NLRP3 inhibitor CRID3/MCC950, providing a structurally characterized native-ligand reference for the holo comparison [21]. Together, these results suggest that the statistically supported Yerba Santa interactions span proteins associated with complementary aspects of neuroinflammatory and neuronal biology; however, experimental studies are required to determine whether these predicted interactions produce measurable effects on receptor, complement, or inflammasome function.

## 4. Materials and Methods

### 4.1. Neuroinflammation-Related Protein Targets and Yerba Santa Compounds

Most of the 30 neuroinflammation-related target structures were based on existing studies [6] and their crystal structures were obtained from the Research Collaboratory for Structural Bioinformatics Protein Data Bank (RCSB PDB). The selection process prioritized co-crystalline structures with bound ligands, ensuring relevance for docking simulations and allowing for native-ligand redocking when applicable. Apo structures were used when an appropriate co-crystallized structure was not available. When multiple structures were available for a given target, the most suitable structure was selected based on two main criteria: resolution and release date. Structures with a resolution better than 2.0 Å were preferred because their higher atomic-level detail improves the accuracy of protein–ligand docking simulations. In addition, more recently resolved structures were prioritized to take advantage of advances in crystallographic methods and data quality. This approach was implemented using the MolSoft Internal Coordinate Mechanics (ICM) platform, which has been extensively benchmarked and validated for accurate protein–ligand pose prediction and for virtual screening across diverse target classes [13,22]. All 30 selected targets were available in the MolSoft ICM64 Pro 3.9 (ICM) [23] docking software database and were prepared for docking analysis (Table 5).

The ligands used in this study were selected based on the chemical characterization of *Eriodictyon* species reported by Wang et al. [7], found in Table 6, which summarizes compounds identified by UHPLC/Q-ToF-MS analysis. These assignments are based on mass spectrometric features (MS peaks) and their corresponding fragmentation patterns rather than definitive structural confirmation. A total of 30 MS peaks were included in the docking analysis. Among these, 24 peaks were classified as unambiguous, for which a single structure could be confidently assigned, whereas 6 peaks were considered ambiguous because more than one structurally plausible candidate could not be distinguished based on MS data alone. For these ambiguous peaks, alternative candidate structures were retained and labeled using suffixes “a” and “b” (e.g., L25a and L25b), indicating mutually exclusive structural hypotheses for the same MS feature rather than distinct co-occurring compounds. Accordingly, ligand numbering reflects MS peak assignments rather than unique chemical entities, and each ambiguous peak was represented by multiple candidate structures evaluated independently, resulting in 36 ligand structures used in docking. Chemical structures were obtained from PubChem when available; for compounds lacking complete structural information, SMILES representations were derived from literature and patent sources, including Makabe et al. [32], with additional validation from phytochemical studies of *Eriodictyon* species [33].

**Table 6:** Molecular Properties and Structures of Yerba Santa Bioactive Compounds (Ligands).

Wang et al. Article_ID	No.	Compound_Name_(Ligand)	Molecular_Formula	MW_(g/mol)	SMILES
6	1	6-methoxynaringenin (5,7,4′-Trihydroxy-6-methoxyflavanone)	C_16_H_14_O_6_	302.28	COC1=C(C2=C(C=C1O)OC(CC2=O)C3=CC=C(C=C3)O)O
42	2	Acacetin (flavone)	C_16_H_12_O_5_	284.26	COC1=CC=C(C=C1)C2=CC(=O)C3=C(C=C(C=C3O2)O)O
24	3	Apigenin (flavone)	C_15_H_10_O_5_	270.24	C1=CC(=C(C=C1C2=CC(=O)C3=C(C=C(C=C3O2)O)O)O)O
17	4	Astragalin	C_21_H_20_O_11_	448.4	C1=CC(=CC=C1C2=C(C(=O)C3=C(C=C(C=C3O2)O)O)O[C@H]4[C@@H]([C@H]([C@@H]([C@H](O4)CO)O)O)O)O
40	5	Chrysin (dihydroxy (flavone)	C_15_H_10_O_4_	254.24	C1=CC=C(C=C1)C2=CC(=O)C3=C(C=C(C=C3O2)O)O
34	6	Cirsimaritin (flavone)	C_17_H_14_O_6_	314.29	COC1=C(C(=C2C(=C1)OC(=CC2=O)C3=CC=C(C=C3)O)O)OC
4	7	Eriodictyol (flavanone)	C_15_H_12_O_6_	288.25	C1[C@H](OC2=CC(=CC(=C2C1=O)O)O)C3=CC(=C(C=C3)O)O
43–44	8	Eriolic Acid A	C_23_H_32_O_5_	388.5	CC(=CCC1=CC(=C(C=C1C(=O)O)O)CCC=C(C)C)C(=O)C
12	9	Eriolic Acid C (bisprenylated benzoic acid)	C_22_H_30_O_5_	374.47	CC(=CCC(C(=CCC1=CC(=CC(=C1O)CC=C(C)CO)C(=O)O)C)O)C
52–53	10	Eriolic Acid D	C_23_H_32_O_4_	372.5	CC(=CCC1=CC(=C(C=C1C(=O)O)O)CCC=C(C)C)C(=O)C
41	11	Genkwanin (flavone)	C_16_H_12_O_5_	284.26	COC1=CC(=C2C(=C1)OC(=CC2=O)C3=CC=C(C=C3)O)O
11	12	Hesperetin (flavanone)	C_16_H_14_O_6_	302.28	COC1=C(C=C(C=C1)[C@@H]2CC(=O)C3=C(C=C(C=C3O2)O)O)O
8	13	Hispidulin (6-methoxyapigenin) (flavone)	C_16_H_12_O_6_	300.26	COC1=C(C2=C(C=C1O)OC(=CC2=O)C3=CC=C(C=C3)O)O
9	14	Homoeriodictyol (flavanone)	C_16_H_14_O_6_	302.28	COC1=C(C=CC(=C1)[C@@H]2CC(=O)C3=C(C=C(C=C3O2)O)O)O
16	15	Isoquercetin (Quercetin 3′-O-glucoside )	C_21_H_20_O_12_	464.38	C1=CC(=C(C=C1C2=C(C(=O)C3=C(C=C(C=C3O2)O)O)O[C@H]4[C@@H]([C@H]([C@@H]([C@H](O4)CO)O)O)O)O)O
10	16	Jaceosidin (trihydroxyflavone)	C_17_H_14_O_7_	330.29	COC1=C(C=CC(=C1)C2=CC(=O)C3=C(O2)C=C(C(=C3O)OC)O)O
5	17	Luteolin (flavone)	C_15_H_10_O_6_	286.24	C1=CC(=C(C=C1C2=CC(=O)C3=C(C=C(C=C3O2)O)O)O)O
3	18	Melitric acid A (salvianolic acid I) (polylhenolic acid)	C_27_H_22_O_12_	538.5	C1=CC(=C(C=C1CC(C(=O)O)OC(=O)/C=C/C2=CC(=C(C=C2)O/C(=C\C3=CC(=C(C=C3)O)O)/C(=O)O)O)O)O
7	19	Naringenin (flavanone)	C_15_H_12_O_5_	272.25	C1[C@H](OC2=CC(=CC(=C2C1=O)O)O)C3=CC=C(C=C3)O
15	20	Pinocembrin (flavanone)	C_15_H_12_O_4_	256.25	C1[C@H](OC2=CC(=CC(=C2C1=O)O)O)C3=CC=CC=C3
1	21	Rosmarinic acid (phenolic acid)	C_18_H_16_O_8_	360.3	C1=CC(=C(C=C1C[C@H](C(=O)O)OC(=O)/C=C/C2=CC(=C(C=C2)O)O)O)O
14	22	Sakuranetin (flavanone)	C_16_H_14_O_5_	286.28	COC1=CC(=C2C(=O)C[C@H](OC2=C1)C3=CC=C(C=C3)O)O
2	23	Salvianolic acid H (3′-O-(8″-Z-Caffeoyl)Rosmarinic Acid)	C_27_H_22_O_12_	538.5	C1=CC(=C(C=C1C[C@H](C(=O)O)OC(=O)/C=C/C2=CC(=C(C=C2)O)O/C(=C\C3=CC(=C(C=C3)O)O)/C(=O)O)O)O
13	24	Sterubin (7-O-Methyleriodictyol) (flavonoid)	C_16_H_14_O_6_	302.28	COC1=CC(=C2C(=O)C[C@H](OC2=C1)C3=CC(=C(C=C3)O)O)O
26–28	25a	Chrysoeriol (5,7-dihydroxy-2-(4-hydroxy-3-methoxyphenyl)chromen-4-one) (flavone)	C_16_H_12_O_6_	300.26	COC1=C(C=CC(=C1)C2=CC(=O)C3=C(C=C(C=C3O2)O)O)O
26–28	25b	Diosmetin (5,7-dihydroxy-2-(3-hydroxy-4-methoxyphenyl)chromen-4-one)	C_16_H_12_O_6_	300.26	COC1=C(C=C(C=C1)C2=CC(=O)C3=C(C=C(C=C3O2)O)O)O
22/23	26a	Eupafolin (Nepetin,6-methoxyluteolin)	C_16_H_12_O_7_	316.26	COC1=C(C2=C(C=C1O)OC(=CC2=O)C3=CC(=C(C=C3)O)O)O
22/23	26b	Isorhamnetin (Flavonol)	C_16_H_12_O_7_	316.26	COC1=C(C=CC(=C1)C2=C(C(=O)C3=C(C=C(C=C3O2)O)O)O)O
31/32	27a	5,4′-Dihydroxy-6,7-Dimethoxyflavanone	C_17_H_14_O_6_	316.3	COC1=C(C(=C2C(=O)CC(OC2=C1)C3=CC=C(C=C3)O)O)OC
31/32	27b	6-Methoxysakuranetin	C_17_H_16_O_6_	316.3	COC1=C(C(=C2C(=O)C[C@H](OC2=C1)C3=CC=C(C=C3)O)O)OC
35/36	28a	Pectolinarigenin (5,7-Dihydroxy-6-methoxy-2-(4-methoxyphenyl)-4-benzopyrone)	C_17_H_14_O_6_	314.29	COC1=CC=C(C=C1)C2=CC(=O)C3=C(O2)C=C(C(=C3O)OC)O
35/36	28b	Velutin (flavone)	C_17_H_14_O_6_	314.29	COC1=CC(=C2C(=C1)OC(=CC2=O)C3=CC(=C(C=C3)O)OC)O
37–39	29a	Erionic Acid C	C_22_H_30_O_5_	374.5	CC(CCc1cc(cc(CC=C(C)CO)c1O)C(O)=O)C(CC=C(C)C)=O
37–39	29b	Erionic Acid D	C_22_H_30_O_5_	374.5	CC(CCc1cc(cc2CC(C(C)(C)Oc12)O)C(O)=O)C(CC=C(C)C)=O
45–48	30a	Eriolic Acid B	C_22_H_30_O_4_	358.47	CC(=CCC1=CC(=C(C=C1C(=O)O)O)CCC=C(C)C)C(=O)C
45–48	30b	Erionic Acid F	C_22_H_30_O_4_	358.47	[H]CC(C)=CCc1cc(cc(CC=C(C)C(CC=C(C)C)=O)c1O)C(O)=O

### 4.2. Target and Ligand Preparation for Docking

A structure data file (SDF) containing 36 bioactive ligands structures representing 30 mass spectrometry (MS) peak assignments reported for Yerba Santa (hereafter referred to as ligands) was prepared to enable docking against the 30 selected protein structures (hereafter referred to as targets). Ligands were processed using the default ICM docking preparation parameters. Charge groups were set to “none”; Keep carboxyls neutral, Sample racemic centers, and Sample double-bond cis/trans were disabled; Relax covalent geometry and Fix peptide-like amides were enabled; and the flexible ring sampling level was set to 2. Alternative tautomeric forms were not systematically enumerated; therefore, the tautomeric forms represented in the input ligand structures were retained for docking. Prior to docking, each target PDB structure underwent standard preparation steps, including deletion of solvent molecules unless they mediated target–ligand interactions or were part of conserved binding-site features. Structure-based preparation procedures automatically retained tightly bound water molecules that contribute to stable interaction networks, including those characteristic of conserved catalytic pockets. Metal ions such as Zn^2+^, Fe^2+^, and Ca^2+^ were deleted unless they were clearly located in the active site, and glycosylation groups such as *N*-acetylglucosamine (NAG) were removed unless structural inspection or annotation in the PDB entry, Universal Protein Resource (UniProt) record, or the associated literature indicated direct involvement in ligand binding or functional relevance for substrate recognition, stabilization, or catalysis.

Two types of target PDB files were processed: holo structures containing co-crystallized ligands and apo structures without co-crystallized ligands. For holo structures, the ligand was first isolated to identify the original binding site, which served as a reference (template) for docking pocket selection. The pocket corresponding to the original ligand was selected, as it represents an experimentally observed ligand-binding site. For apo structures, binding pockets were inferred through structure-based pocket analysis, using the ICM Pocket Finder tool, and pocket volume, hydrophobic character, and buriedness were used to evaluate their suitability for ligand binding. The pocket with the highest DLID and a volume within the preferred range of 300–600 Å^3^ (not exceeding 800 Å^3^) was selected for docking.

For conventional pose validation of holo structures, the corresponding crystallographic ligand was redocked into its experimentally defined binding site using the crystallographic ligand as the docking template. Redocking was performed for 100 independent runs for each holo structure. Because the redocked ligand and the crystallographic reference represented the same molecule, pose recovery was evaluated by RMSD, with values <0.5 Å indicating successful reproduction of the experimental binding orientation. The resulting native-ligand docking score distributions were also retained as target-specific reference distributions for subsequent comparison with the Yerba Santa ligand docking scores. This RMSD validation was not applicable to apo structures because no crystallographic ligand pose was available as a reference, or to comparisons between Yerba Santa ligands and the crystallographic ligands because they represent different molecular structures and therefore lack an equivalent atom-to-atom reference for conventional pose comparison.

**Figure 9:**
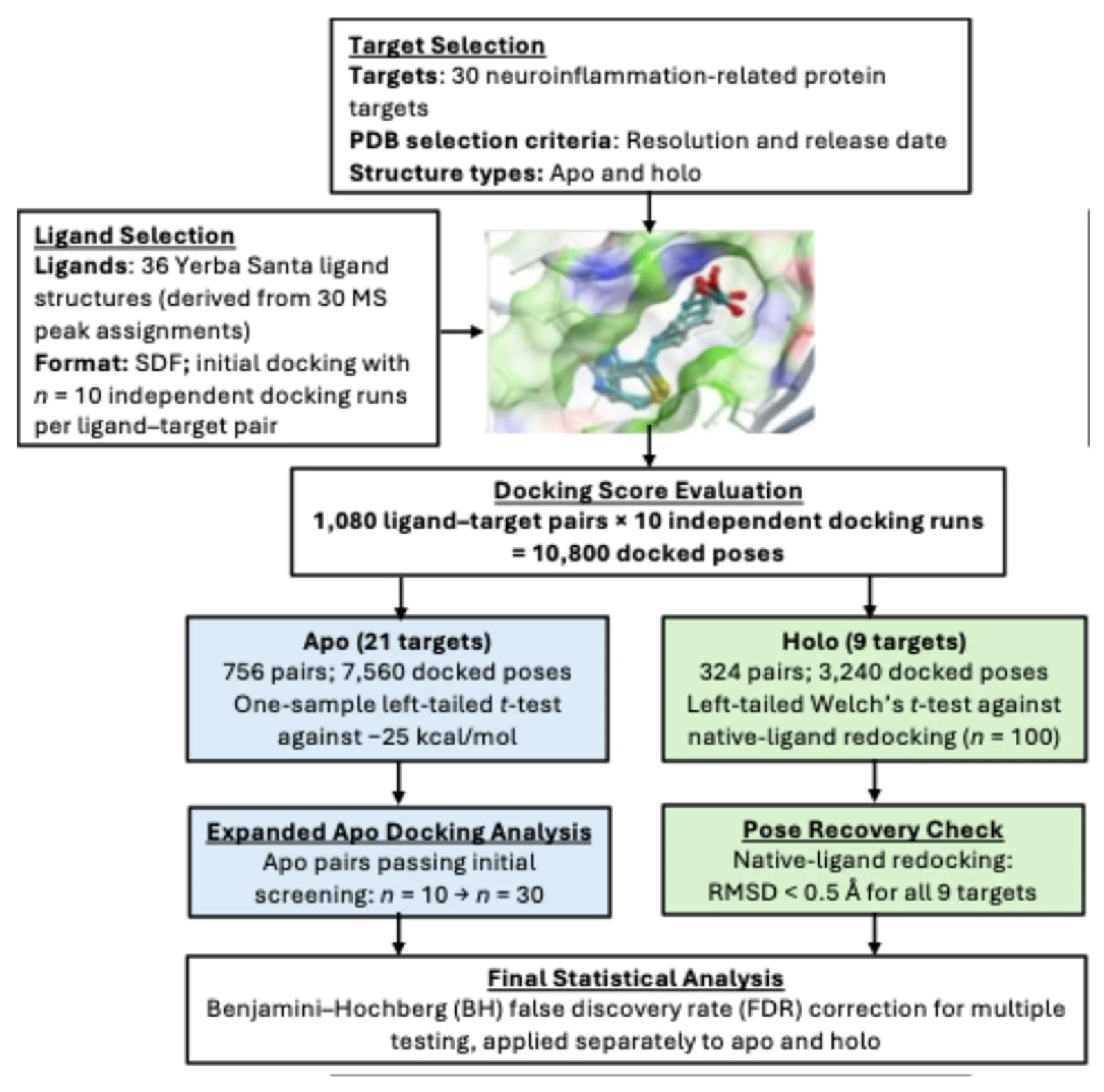
A schematic representation of the docking and validation pipeline used to screen 36 Yerba Santa ligand structures (corresponding to 30 MS peak assignments) [7] across 30 neuroinflammation-associated protein targets [15]. Initial docking consisted of *n* = 10 independent runs per ligand–target pair. For the 9 holo targets, native ligands were redocked (*n* = 100) to provide target-specific reference distributions for comparison with Yerba Santa ligand docking scores. The 21 apo targets were screened against the −25 kcal/mol criterion, with selected pairs expanded to *n* = 30. Benjamini–Hochberg false discovery rate (BH-FDR) correction was applied separately to the apo and holo multiple-testing analysis.

A schematic overview of the complete docking and validation workflow, including target preparation, initial docking analysis, native-ligand redocking and pose validation for holo structures, expanded *n* = 30 docking analysis for selected apo ligand–target pairs, and post-docking statistical evaluation, is shown in Figure 9.

### 4.3. Docking Score Evaluation and Interpretation

The ICM scoring method uses a GBSA/MM-type energy function, which combines molecular mechanics (MM) with an implicit solvation model that approximates water effects. MM accounts for the internal energy of the system through bond, angle, torsion, van der Waals, and electrostatic interactions. GBSA (Generalized Born Surface Area) estimates solvation free energy by adding a polar contribution, calculated with the Generalized Born model to capture electrostatic effects, and a nonpolar contribution, derived from the solvent-accessible surface area (SASA) to approximate nonpolar solvation effects. The scoring function also includes a directional hydrogen bonding term, which evaluates both the strength and geometry of hydrogen bonds, rewarding short, nearly linear donor-hydrogen-acceptor interactions [13]. While the docking score provides a computational metric for ranking predicted ligand–target interactions, its main purpose in virtual ligand screening (VLS) is to facilitate discrimination between compounds with more and less favorable predicted interactions rather than to reproduce exact binding energies.

The ICM docking score for each docked conformation was computed using the following scoring function [23]:

ΔG = C_1_ΔE_HBond + C_2_ΔE_Vwlnt + C_3_ΔE_HPhob + C_4_ΔE_Solv + C_5_ΔE_SolEl + C_6_ΔE_Intl + C_7_TΔSSc + C_8_Qatom (1)

where the individual terms represent hydrogen bond energy (ΔE_HBond), van der Waals interaction energy (ΔE_Vwlnt), hydrophobic interaction energy (ΔE_HPhob), desolvation energy (ΔE_Solv), solvation electrostatic energy (ΔE_SolEl), internal molecular energy (ΔE_Intl), conformational entropy loss (TΔSSc), and a correction for the number of atoms (Qatom). C_i_ is the coefficient determined by ICM. The resulting ΔG-based score combines these energetic contributions to estimate the relative favorability of a predicted ligand–target interaction within the ICM scoring model. It should not be interpreted as an experimentally determined or absolute thermodynamic binding free energy [23].

More negative docking scores indicate more favorable predicted ligand–target interactions within the ICM scoring model, reflecting improved geometric complementarity and favorable predicted interaction networks within the binding site. These scores do not represent absolute thermodynamic binding free energies but rather provide a relative measure of interaction favorability that can be used to rank ligands docked to the same target under identical conditions. Accordingly, docking scores were used for relative ranking and screening rather than as direct measures of experimental binding affinity or thermodynamic stability.

### 4.4. Docking Output and Score Distribution Assessment

A total of 36 ligands (corresponding to 30 MS peak assignments) were docked against 30 protein targets, generating 1080 ligand–target combinations. For each combination, *n* = 10 independent docking runs were initially performed per ligand–target pair, using an SDF containing 10 repetitions, resulting in 10,800 total docked poses during the primary screening stage. Docking was conducted in MolSoft ICM with Store Alternative Conformations disabled and Max Number of Conformations set to 1, ensuring one pose per run. The Monte Carlo-based sampling algorithm was employed to mimic stochastic perturbations of ligand translational, rotational, and internal coordinates, followed by local optimization within precomputed grid potential maps.

Consequently, individual docking runs represent independent stochastic realizations rather than deterministic optimizations. A conventional single-run docking analysis therefore represents only one realization of this stochastic procedure and could, in principle, correspond to any individual result within the distribution observed across independent repeated docking runs. Variability in docking scores across repeated runs therefore reflects differences in sampled binding modes and local energy minima, rather than numerical instability of the scoring function. Evaluation of docking score distributions across multiple independent runs, rather than reliance on a single best-scoring pose, provides a more representative characterization of docking score behavior and reproducibility, consistent with established ICM benchmarking studies [13]. In particular, Masuda et al. demonstrated that histogram-based learning models constructed from distributions of multiple docking scores outperform single-score approaches, indicating that ensembles of docking poses capture informative structural and energetic features relevant to protein–ligand binding [14].

Docking scores obtained from the initial *n* = 10 simulations were evaluated according to target structure type. For the 9 holo targets, native crystallographic ligands were redocked using *n* = 100 independent runs, and the resulting native-ligand docking score distributions were used as target-specific reference distributions for comparison with Yerba Santa ligand docking scores. For the 21 apo targets, for which native-ligand reference distributions were unavailable, docking scores were evaluated using one-sample, left-tailed Student’s *t*-tests against the statistical reference value of −25 kcal mol^−1^. Apo ligand–target pairs passing the initial screening were expanded from *n* = 10 to *n* = 30 and re-evaluated using the same one-sample, left-tailed Student’s *t*-test against the statistical reference value of −25 kcal mol^−1^. To account for multiple testing, Benjamini–Hochberg false discovery rate (FDR) correction was applied to the *p*-values obtained from the 756 initial *n* = 10 apo ligand–target comparisons.

Given this stochastic sampling framework, additional analyses were performed to assess whether the number of docking repetitions was sufficient to capture stable score distributions. To evaluate whether increasing the number of docking repetitions (represented by *n*) would materially alter the distributional features observed with this design, one representative ligand–target pair was additionally subjected to expanded docking ensembles consisting of *n* = 100 and *n* = 500 independent dockings. These additional docking runs were used solely to evaluate the stability of score distributions with respect to increased sampling density and to support the adequacy of the *n* = 10 initial sampling framework used in this study. Histograms of docking scores were used to assess the overall docking score landscape and score distribution for each target across all screened ligands. This analysis was used to visualize whether a receptor produced more or less favorable predicted docking scores across the screened ligands and whether the resulting score distributions were unimodal or bimodal, reflecting differences in docking score behavior among the screened compounds.

### 4.5. Structure-Dependent Docking Score Reference Criteria and Statistical Evaluation

To address the limitations associated with applying a universal docking score cutoff to structurally diverse protein targets, the 30 selected protein structures were classified into apo (*n* = 21) and holo (*n* = 9) categories according to the availability of a co-crystallized native ligand. Distinct docking score reference criteria and statistical procedures were applied to these two categories. For apo targets, the predefined ICM docking score criterion of −25 kcal/mol was retained for initial screening and prioritization. For holo targets, no universal docking score cutoff was imposed; instead, the corresponding crystallographic native ligands were redocked to generate target-specific computational reference distributions for comparison with the Yerba Santa ligands. This structure-dependent approach allowed for the use of experimentally observed ligand-binding sites as computational references where available while retaining a consistent screening procedure for apo structures without such references.

For apo structures, an ICM docking score of ≤−25 kcal/mol was used as a predefined computational screening criterion. This criterion was based on MolSoft guidance for virtual ligand screening [23], which indicates that compounds with scores weaker than −25 are generally not considered worth further evaluation, except in low-affinity fragment screening. MolSoft further indicates that scores better than −30 are generally expected for most pockets, with large or deeply buried pockets often yielding scores beyond −40. Accordingly, the −25 criterion was used as a pass/fail screening criterion for prioritization rather than as an experimentally validated threshold for binding affinity or thermodynamic stability [23]. Docking scores were exported and processed in MATLAB_R2024b to group and compute the mean and standard deviation across the initial *n* = 10 runs per ligand–target pair. Prior to statistical testing, score distributions were inspected using histogram analysis to visualize the overall docking score landscape and assess convergence across different numbers of runs. For a representative ligand–target system, additional docking ensembles (*n* = 100 and *n* = 500 runs) were generated and compared with the standard *n* = 10 sampling to verify that the distribution shape, central tendency, and variance remained stable as sampling increased. Histogram plots were generated using 50 bins, and peak positions were estimated by identifying the bin with the highest frequency. Mean docking scores were indicated by vertical reference lines, and a cutoff of −25 kcal mol^−1^ was included to indicate the predefined ICM screening criterion.

For the 21 apo targets, to assess whether individual ligand–target combinations had mean docking scores statistically below the statistical reference value across multiple runs, a one-sample, left-tailed Student’s *t*-test was performed in MATLAB. For each pair, the sample mean ($\overset{¯}{x}$) and standard deviation (s) were calculated. The test statistic was computed as t = ($\overset{¯}{x}$ − μ_0_)/(s/√*n*), where *n* = 10 and μ_0_ = −25 kcal mol^−1^, representing the statistical reference value used in the analysis. The null hypothesis (H_0_: μ = −25 kcal mol^−1^) was tested against the alternative (H_1_: μ < −25 kcal mol^−1^). To account for multiple testing, Benjamini–Hochberg false discovery rate (BH-FDR) correction was applied to the *p*-values obtained from the 756 initial *n* = 10 apo ligand–target comparisons. Only apo ligand–target pairs remaining statistically significant after BH-FDR correction (q < 0.05) were included in the *n* = 30 follow-up analysis and re-evaluated using the same one-sample, left-tailed Student’s *t*-test against the statistical reference value of −25 kcal mol^−1^. BH-FDR correction was then applied separately to the *p*-values obtained from the *n* = 30 follow-up comparisons, with q < 0.05 considered statistically significant.

For the 9 holo targets, the −25 kcal mol^−1^ statistical reference value was not applied. Instead, Yerba Santa ligand docking score distributions (*n* = 10) were compared with the corresponding target-specific native-ligand docking scores obtained from *n* = 100 redocking runs. Statistical comparisons were performed within each target using a left-tailed Welch’s two-sample *t*-test, to test whether the mean Yerba Santa ligand docking score was lower than the mean native-ligand docking score. Benjamini–Hochberg false discovery rate (BH-FDR) correction was applied across all 324 holo ligand–target comparisons, with q < 0.05 considered statistically significant. These comparisons were not interpreted as direct measures of experimental binding affinity. By comparing each Yerba Santa ligand with the corresponding native-ligand docking score distribution within the same protein structure, the holo target analysis avoided the application of an identical numerical screening cutoff across structurally distinct protein targets. These comparisons establish relative computational scoring behavior within each target and do not demonstrate experimentally stronger binding or functional activity.

Separately, to quantitatively evaluate convergence between different sampling depths, Welch’s two-sample *t*-tests were performed to compare mean docking scores obtained from *n* = 10 runs with those obtained from larger ensembles (*n* = 100 and *n* = 500). This test was selected because it does not assume equal variances between groups. The test statistic, *p*-value, and 95% confidence intervals were calculated, and effect size was estimated using Hedges’ g, a standardized effect size that quantifies the magnitude of the difference between group means in units of standard deviation. Unlike *p*-values, which indicate whether a difference is statistically significant, Hedges’ g provides a measure of the practical magnitude of that difference [34]. In this context, its use complements Welch’s *t*-test by enabling assessment of whether differences between sampling depths (*n* = 10 vs. *n* = 100 and *n* = 500) are not only statistically significant but also practically meaningful. These comparisons provided statistical support for evaluating whether the standard sampling depth (*n* = 10) adequately represents the underlying docking score distribution. For the apo ligand–target pairs selected during initial screening, the expanded *n* = 30 docking simulations provided an additional assessment of the reproducibility of docking score behavior with increased sampling.

Thus, apo and holo targets were evaluated using separate target-appropriate procedures: apo ligand–target pairs were prioritized using the predefined screening criterion and statistical reference value, whereas holo ligand–target pairs were evaluated relative to their corresponding native-ligand docking score distributions. The apo test evaluates scores against the predefined −25 kcal/mol reference, whereas the holo test compares scores with a target-specific redocked native ligand. Consequently, their *p*-values and BH-FDR-adjusted q-values have different interpretations and cannot be pooled or used to rank pairs across the apo and holo categories.

### 4.6. Structural Characterization of Yerba Santa Ligands

Structural characterization of Yerba Santa ligands was performed to support the interpretation of docking results. Each ligand was analyzed using its SMILES notation and corresponding two-dimensional molecular structure to identify scaffold class, functional groups, stereochemical features, and predicted interaction behavior (Table 7). This systematic structural characterization provides the basis for interpreting the ligand–target interaction patterns observed in the docking results and validation analyses. Ligands were grouped into major structural families based on their core ring systems and dominant substituents. The primary scaffold classes identified were flavones, flavanones, polyphenolic acids, prenylated benzoic acids, flavonol glycosides, and phenylacrylates. Functional groups were annotated directly from each ligand’s SMILES string and 2D representation, providing insight into their potential protein–ligand interactions. Annotated groups included phenolic hydroxyls, carboxylates, carbonyls, methoxy substituents, prenyl chains, glycosyl moieties, and aromatic rings. Aromatic systems were consistently encoded using Kekulé notation (alternating double bonds), rather than the lowercase ‘c’ aromatic representation.

Stereochemical analysis focused on the presence and positioning of chiral centers, particularly in flavanones. These ligands exhibit a saturated C2–C3 bond, with the C2 atom acting as a stereogenic center, often appearing as [C@@H] in the SMILES format. This tetrahedral geometry, absent in flavones, introduces 3D non-planarity that may facilitate adaptation to curved or partially buried binding pockets. Natural flavanones typically adopt the (2S) configuration, which may influence docking orientation compared with their planar flavone counterparts. Predicted interaction capabilities were inferred from the functional group composition using established rules of protein–ligand recognition. Phenolic hydroxyls and carbonyl groups were expected to participate in hydrogen bonding, while carboxylates may form salt bridges and contribute to electrostatic attraction. Aromatic systems may facilitate π-π stacking and participate in favorable cation–π interactions with nearby cationic side chains, particularly Arg and Lys, when appropriately positioned within the binding site [35]. Prenyl chains may facilitate hydrophobic contacts in nonpolar cavities, and glycosyl groups provided multiple hydrogen-bond donors and acceptors, potentially favoring interactions in highly polar pockets. Together, these features may contribute to the diversity of predicted interactions observed across scaffolds.

Docking scores were interpreted in the context of the annotated structural and interactional features of each ligand. More negative scores indicated more favorable predicted interactions within the ICM scoring model and could be associated with multiple favorable contacts, including hydrogen bonds, π-π stacking, electrostatic interactions, and hydrophobic packing. Ligands with higher functional group density or increased conformational flexibility, such as polyphenolic acids and glycosylated flavonoids, were evaluated for their potential to form multiple interaction types within protein-binding pockets. For apo targets, ligand–target pairs passing the initial *n* = 10 screening were further evaluated using the expanded *n* = 30 docking analysis. For holo targets, Yerba Santa ligand docking score distributions were evaluated relative to the corresponding target-specific native-ligand distributions obtained from *n* = 100 redocking runs. These considerations provide a structural framework for interpreting docking results as a function of both the chemical composition and spatial adaptability of the ligands.

## 5. Conclusions, Limitations and Future Directions

Alzheimer’s disease involves multiple interacting pathological processes, including neuroinflammation, complement activation, oxidative stress, mitochondrial dysfunction, and synaptic injury. In this study, we developed an integrated structure–statistical docking framework to evaluate chemically characterized Yerba Santa compounds against neuroinflammation-related protein targets relevant to Alzheimer’s disease. By combining replicated molecular docking with statistical assessment, this approach reduced reliance on single docking scores and prioritized ligand–target pairs with reproducible predicted binding behavior. An important methodological feature of this study was the adoption of a structure-dependent docking score reference strategy rather than the application of a universal −25 kcal/mol cutoff across all protein targets. By distinguishing between apo and holo structures, the framework retained a predefined computational screening criterion for targets without co-crystallized native ligands while incorporating target-specific native-ligand redocking references for ligand-bound structures. The separate apo and holo analyses retained 11 and 23 ligand–target pairs, respectively, under their different statistical criteria. Among the apo targets, 11 ligand–target pairs involving C1r-C1s (PDB: 6F39) remained statistically significant after Benjamini–Hochberg false discovery rate (BH-FDR) correction and expanded docking analysis (*n* = 30). Among the holo targets, 23 ligand–target pairs remained statistically significant after BH-FDR correction, comprising seven interactions with complement C1q (PDB: 2JG8), 15 with GABA-A receptor α1β2γ2 (PDB: 6D6U), and one with NLRP3 (PDB: 7PZC). These statistically supported interactions involved multiple Yerba Santa compounds, predominantly flavonoids, whereas Rosmarinic acid was retained for both complement C1q and NLRP3. These findings suggest that specific Yerba Santa compounds may exhibit preferential predicted interactions with selected protein targets rather than interact broadly across the entire screened protein set.

From a biological perspective, the prioritized ligand–target interactions suggest that Yerba Santa compounds may interact with proteins involved in biological pathways relevant to neuroinflammation and neurodegeneration. The convergence of multiple flavonoids on C1r-C1s and the identification of additional statistically supported interactions with complement C1q highlight complement signaling as a potential pathway for further investigation. The interactions involving GABA-A receptor α1β2γ2 and NLRP3 extend the computational findings to proteins associated with inhibitory neurotransmission and inflammasome-mediated inflammatory responses, respectively. Together, these results provide a rationale for further investigation of the potential involvement of Yerba Santa compounds in multiple biological processes associated with AD. However, the computational results do not establish whether these interactions alter target function or contribute to the reported biological effects of Yerba Santa.

Several limitations should be noted. Molecular docking cannot confirm physiological binding, functional inhibition, cellular activity, or therapeutic efficacy. Docking scores are also influenced by protein structure quality, pocket selection, ligand preparation, and scoring function assumptions. Moreover, the apo and holo *p*-values and q-values test different reference comparisons; they are not equivalent measures of evidence across targets. The concentration of retained pairs among four targets may partly reflect these reference differences and target-specific scoring behavior. Furthermore, statistical significance across replicated docking runs indicates reproducibility of docking score behavior within the applied computational framework but does not establish experimental binding affinity, functional inhibition, or biological efficacy. Accordingly, the statistically supported interactions identified in this study should be interpreted as hypotheses for experimental investigation rather than predictions of therapeutic efficacy. Although native-ligand redocking provided target-specific computational references for holo structures, these reference distributions do not constitute experimentally validated active and inactive compound learning sets. Therefore, the ability of the revised docking framework to discriminate experimentally confirmed binders from nonbinders remains to be established.

To further assess the biological relevance of the prioritized interactions, experimentally characterized reference compounds and site-matched experimental binding data should be considered for the four retained targets. For C1r-C1s, the protein structure used in the apo analysis (PDB: 6F39) represents the CUB1-EGF-CUB2 region of C1r rather than its C-terminal catalytic serine-protease domain [36]. Therefore, inhibitory data obtained using the catalytic domain cannot be used directly to validate the docking scores obtained for the modeled CUB1-EGF-CUB2 region. For the three retained holo targets, native-ligand redocking provided target-specific computational reference distributions and enabled evaluation of crystallographic pose recovery. However, comparisons with the corresponding native-ligand docking scores do not establish experimentally stronger binding or functional modulation by the Yerba Santa compounds. Further experimental studies using appropriately characterized reference compounds and target-specific binding assays will be required to assess the biological significance of these predicted interactions.

An additional limitation is that the pharmacokinetic and pharmacodynamic properties of the prioritized compounds were not evaluated. Properties such as aqueous solubility, metabolic stability, bioavailability, and blood–brain barrier penetration may substantially influence whether these compounds can reach their proposed molecular targets at biologically relevant concentrations. Consequently, the potential contributions of central and peripheral mechanisms to their biological activities remain unclear.

Future studies should experimentally evaluate the prioritized ligand–target interactions using complementary biochemical, biophysical, and cell-based approaches. Direct binding assays should first be performed, where technically feasible, to determine whether the prioritized compounds interact with their proposed targets and to characterize their binding affinities. For the complement-related targets, biochemical complement assays and appropriate functional studies should determine whether the predicted interactions with the C1r CUB1-EGF-CUB2 region or complement C1q affect complement activation or other associated functions. In particular, the potential effects of ligand interactions with the C1r CUB1-EGF-CUB2 region should be investigated without assuming direct inhibition of the catalytic serine-protease domain. For GABA-A receptor α1β2γ2, receptor-binding and functional assays should investigate whether the prioritized compounds alter receptor activity. For NLRP3, direct binding and inflammasome-related functional assays should evaluate whether rosmarinic acid interacts with the target and produces measurable effects on NLRP3-associated inflammatory signaling. These studies will help distinguish predicted structural interactions from functionally meaningful target modulation.

Subsequent cell-based studies should evaluate whether the prioritized compounds reach their proposed targets under biologically relevant conditions and produce measurable effects on neuroinflammatory pathways or neuronal injury. Human cell-based models relevant to neuroinflammation and neuronal function could be used to investigate the effects of the prioritized compounds on complement-associated inflammatory responses, GABA-A receptor signaling, and NLRP3 inflammasome activation, as appropriate for each target. In parallel, ADMET evaluation and blood–brain barrier permeability studies will be important for assessing compound exposure, identifying potential pharmacokinetic limitations, and determining whether central or peripheral mechanisms are more plausible.

Overall, this study provides a reproducible computational framework for prioritizing Yerba Santa ligand–target interactions and identifies ligand–protein systems involving C1r-C1s, complement C1q, GABA-A receptor α1β2γ2, and NLRP3 for target-specific follow-up investigation. The findings highlight the potential value of integrating replicated molecular docking with structure-dependent reference criteria and statistical assessment to generate experimentally testable hypotheses regarding natural-product interactions with neuroinflammation-related proteins. Nevertheless, experimental confirmation of target engagement, functional modulation, cellular activity, and pharmacokinetic properties will be necessary before the potential therapeutic relevance of these interactions to AD can be established.

## Figures and Tables

**Table 1:** ICM docking scores for 36 Yerba Santa natural ligands on two of the 30 neuroinflammation targets: Arf6 (T1; PDB:1E0S, holo, with co-crystallized ligand) and C1r-C1s (T23; PDB:6F39, apo, without co-crystallized ligand), showing ICM scores across 10 independent docking runs. Green highlighting indicates apo docking scores ≤−25 kcal/mol. The −25 kcal/mol screening criterion applies only to the apo target analysis, whereas holo target scores are evaluated relative to the corresponding native-ligand computational reference distributions.

	Target No.	1										23									
	Target Name	1E0S (Holo, with Ligand)										6F39 (Apo, Without Ligand)									
Ligand No.	Ligand Name	RUN1	RUN2	RUN3	RUN4	RUN5	RUN6	RUN7	RUN8	RUN9	RUN10	RUN1	RUN2	RUN3	RUN4	RUN5	RUN6	RUN7	RUN8	RUN9	RUN10
**1**	6-methoxynaringenin (5,7,4′-Trihydroxy…	−10	−10	−8	−8	−13	−7	−8	−8	−10	−10	−25	−23	−23	−21	−21	−24	−25	−24	−25	−25
**2**	Acacetin (flavone)	−16	−17	−16	−16	−18	−18	−18	−17	−7	−18	−20	−19	−20	−20	−29	−26	−26	−20	−29	−29
**3**	Apigenin (flavone)	−16	−20	−16	−16	−16	−16	−16	−16	−16	−16	−26	−27	−27	−27	−27	−27	−27	−27	−27	−27
**4**	Astragalin	−5	−5	−4	−4	−4	−1	−4	−9	−8	−8	−19	−3	−17	−14	−18	−15	−18	−12	−15	−12
**5**	Chrysin (dihydroxy (flavone)	−18	−18	−18	−18	−17	−18	−18	−18	−18	−18	−28	−28	−28	−30	−28	−28	−28	−28	−28	−28
**6**	Cirsimaritin (flavone)	−10	−10	−12	−10	−10	−10	−8	−10	−10	−10	−26	−26	−29	−27	−26	−29	−27	−29	−25	−26
**7**	Eriodictyol (flavanone)	−15	−15	−12	−15	−12	−12	−9	−12	−10	−13	−26	−26	−27	−25	−27	−22	−27	−27	−27	−26
**8**	Eriolic Acid A	−13	−13	−19	−15	−13	4	−19	−19	−4	−17	−7	−3	−12	−15	−4	−13	−17	−3	−7	27
**9**	Eriolic Acid C (bisprenylated benzoic acid)	−14	−17	−12	−13	−11	−2	−11	−13	−11	−12	−6	−10	−10	−3	−8	−17	−3	−10	−10	18
**10**	Eriolic Acid D	−17	−8	−17	−11	−8	−17	−9	−8	−17	−16	−13	−15	−13	−16	−22	−22	−19	−21	−9	−14
**11**	Genkwanin (flavone)	−11	−18	−18	−16	−9	−16	−9	−18	−15	−18	−26	−28	−28	−28	−33	−28	−26	−26	−27	−28
**12**	Hesperetin (flavanone)	−17	−4	−15	−11	−14	−14	−7	−6	−15	−12	−27	−23	−19	−24	−22	−27	−20	−13	−13	−24
**13**	Hispidulin (6-methoxyapigenin) (flavone)	−6	−17	−10	−17	−16	−8	−16	−17	−17	−16	−28	−28	−27	−27	−27	−27	−28	−28	−28	−24
**14**	Homoeriodictyol (flavanone)	−16	−11	−11	−14	−16	−16	−10	−15	−3	−16	−26	−25	−26	−25	−25	−25	−26	−25	−22	−20
**15**	Isoquercetin (Quercetin 3′-O-glucoside )	−2	−9	−10	−14	−9	−10	−12	−9	2	2	−18	−14	−13	−18	−15	−11	−13	−14	−18	1
**16**	Jaceosidin (trihydroxyflavone)	−17	−13	−17	−16	−17	−17	−17	−17	−17	−17	−27	−28	−26	−19	−20	−31	−28	−21	−28	−24
**17**	Luteolin (flavone)	−16	−16	−15	−17	−16	−16	−19	−16	−17	−16	−27	−27	−27	−27	−27	−27	−27	−27	−25	−27
**18**	Melitric acid A (salvianolic acid I) (pol…	−16	−21	−22	−29	−24	−17	−20	−15	−29	−31	−26	−23	−29	−14	−30	−28	−29	−17	−25	−26
**19**	Naringenin (flavanone)	−15	−15	−15	−15	−15	−15	−15	−15	−15	−16	−24	−20	−26	−26	−24	−26	−26	−26	−28	−26
**20**	Pinocembrin (flavanone)	−15	−15	−15	−15	−15	−15	−15	−15	−15	−15	−28	−29	−28	−28	−28	−28	−29	−28	−29	−29
**21**	Rosmarinic acid (phenolic acid)	−22	−20	−21	−13	−19	−18	−19	−20	−19	−24	−16	−22	−24	−27	−27	−21	−24	−27	−23	−23
**22**	Sakuranetin (flavanone)	−15	−13	−9	−9	−15	−16	−15	−10	−16	−14	−27	−26	−27	−27	−27	−18	−26	−26	−27	−27
**23**	Salvianolic acid H (3′-O-(8″-Z-Caffeoyl…	−31	−35	−30	−36	−9	−34	−38	−20	−26	−16	−22	−22	−22	−21	−9	−20	−26	−20	−14	−21
**24**	Sterubin (7-O-Methyleriodictyol) (flavo…	−9	−14	−15	−15	−8	−10	−12	−12	−14	−12	−27	−27	−28	−27	−28	−27	−27	−25	−28	−28
**25a**	Chrysoeriol (5,7-dihydroxy-2-(4-hydrox…	−10	−11	−16	−11	−18	−13	−12	−17	−17	−14	−29	−30	−26	−27	−30	−29	−24	−29	−30	−28
**25b**	diosmetin (5,7-dihydroxy-2-(3-hydroxy-…	−15	−15	−15	−3	−16	−15	−15	−15	−15	−16	−24	−28	−28	−28	−18	−19	−28	−19	−19	−17
**26a**	Eupafolin (Nepetin,6-methoxyluteolin)	−9	−15	−15	−15	−15	−6	−15	−16	−14	−15	−27	−23	−19	−24	−27	−23	−27	−23	−23	−21
**26b**	Isorhamnetin (Flavonol)	−20	−17	−17	−17	−15	−20	−17	−17	−17	−9	−28	−32	−32	−31	−32	−33	−22	−32	−32	−32
**27a**	5,4′-Dihydroxy-6,7-Dimethoxyflavanone	−7	−7	−9	−6	−8	−7	−9	−6	−6	−9	−28	−27	−27	−22	−27	−28	−27	−28	−28	−27
**27b**	6-Methoxysakuranetin	−7	−5	−8	−9	−7	−5	−7	−10	−8	−8	−27	−27	−28	−28	−28	−27	−26	−27	−28	−28
**28a**	Pectolinarigenin (5,7-Dihydroxy-6-met…	−10	−6	2	1	0	1	1	−13	−13	−4	−29	−27	−29	−28	−28	−20	−29	−26	−27	−26
**28b**	Velutin (flavone)	−10	−10	−14	−11	−11	−10	−10	−18	−11	−10	−34	−32	−34	−34	−34	−30	−34	−34	−34	−34
**29a**	Erionic Acid C	−18	−4	−16	−20	−2	−6	−11	−3	−9	−8	−7	−15	−21	−15	−7	−18	−8	−17	−17	−8
**29b**	Erionic Acid D	−18	−16	−14	−19	−6	−10	−9	−14	−17	−14	−8	−13	−18	−2	2	−7	−7	−7	−3	1
**30a**	Eriolic Acid B	−12	−10	1	−8	−12	−13	−5	−13	−10	−13	−8	−15	−12	−19	−8	−15	−15	−23	−8	19
**30b**	Erionic Acid F	−13	−18	−12	−20	−11	−20	−21	−13	−22	−10	−13	−8	−23	−22	−14	−20	−20	−19	18	−21

**Table 2:** Statistical analysis of the 11 C1r-C1s (PDB:6F39; T23) ligand–target pairs retained after BH-FDR correction in the initial *n* = 10 analysis and re-evaluated at *n* = 30. *p*-values were obtained using a one-sample, left-tailed Student’s *t*-test against −25 kcal mol^−1^; q-values are BH-FDR-adjusted. All 11 interactions remained statistically significant at *n* = 30 (q < 0.05).

Ligand No.	Ligand Name	*n* = 10				*n* = 30			
		Mean	SD	*p*-Value	q-Value	Mean	SD	*p*-Value	q-Value
L20	Pinocembrin	−28.45	0.192	4.12 × 10^−13^	3.12 × 10^−10^	−28.46	0.147	6.95 × 10^−42^	7.65 × 10^−41^
L27b	6-Methoxysakuranetin	−27.663	0.279	1.17 × 10^−10^	4.42 × 10^−8^	−27.695	0.284	1.80 × 10^−30^	9.87 × 10^−30^
L6	Cirsimaritin	−28.121	0.426	1.25 × 10^−9^	3.15 × 10^−7^	−27.03	2.106	5.84 × 10^−6^	6.43 × 10^−6^
L5	Chrysin	−28.27	0.494	3.06 × 10^−9^	5.54 × 10^−7^	−28.411	0.698	2.71 × 10^−22^	9.94 × 10^−22^
L19	Naringenin	−25.807	0.125	3.67 × 10^−9^	5.54 × 10^−7^	−25.755	0.256	2.48 × 10^−16^	5.45 × 10^−16^
L28b	Velutin	−33.197	1.536	2.01 × 10^−8^	2.54 × 10^−6^	−32.88	1.799	5.63 × 10^−21^	1.55 × 10^−20^
L22	Sakuranetin	−26.566	0.309	3.19 × 10^−8^	3.44 × 10^−6^	−26.704	0.836	2.56 × 10^−12^	4.70 × 10^−12^
L26b	Isorhamnetin	−30.776	2.289	1.13 × 10^−5^	1.07 × 10^−3^	−30.575	3.101	4.66 × 10^−11^	6.41 × 10^−11^
L17	Luteolin	−26.634	0.726	2.77 × 10^−5^	2.33 × 10^−3^	−26.45	1.678	2.66 × 10^−5^	2.66 × 10^−5^
L3	Apigenin	−26.307	0.748	1.83 × 10^−4^	1.38 × 10^−2^	−26.583	0.854	2.34 × 10^−11^	3.68 × 10^−11^
L11	Genkwanin	−30.025	3.128	3.31 × 10^−4^	2.28 × 10^−2^	−30.302	3.38	9.14 × 10^−10^	1.12 × 10^−9^

**Table 3:** Summary of statistically supported apo and holo ligand–target interactions.

Structure Type	Analysis Stage	Comparisons	Sampling	Statistical Criterion	Significant Pairs	Targets Evaluated/Retained	Target	Ligands
Apo	Initial analysis	756	*n* = 10	One-sample left-tailed t-test vs. −25; *p* < 0.05	14	T2, T4, T6, T8, T9, T10, T11, T12, T14, T15, T16, T17, T20, T23, T24, T25, T26, T27, T28, T29, T30	N/A	N/A
Apo	BH-FDR correction	756	*n* = 10	BH-FDR across all 756 apo tests; q < 0.05	11	T23	N/A	N/A
Apo	Expanded follow-up	11	*n* = 30	One-sample left-tailed t-test vs. −25; BH-FDR across 11 tests; q < 0.05	11	T23	T23: C1r-C1s (PDB:6F39)	L20, L27b, L6, L5, L19, L28b, L22, L26b, L17, L3, L11
Holo	Initial comparison	324	YS n = 10 vs. native n = 100	Left-tailed Welch two-sample t-test; *p* < 0.05	32	T1, T3, T5, T7, T13, T18, T19, T21, T22	N/A	N/A
Holo	BH-FDR correction	324	YS n = 10 vs. native n = 100	BH-FDR; q < 0.05	23	T7, T21, T13	T7: C1q (PDB:2JG8)	L20, L19, L4, L2, L13, L5, L21
Holo	BH-FDR correction	324	YS n = 10 vs. native n = 100	BH-FDR; q < 0.05	23	T7, T21, T13	T21: GABA-A receptor α1β2γ2 (PDB:6D6U)	L17, L19, L26b, L1, L26a, L13, L25a, L15, L3, L5, L12, L2, L25b, L20, L4
Holo	BH-FDR correction	324	YS n = 10 vs. native n = 100	BH-FDR; q < 0.05	23	T7, T21, T13	T13: NLRP3 (PDB: 7PZC)	L21

**Table 4:** Structural classification, key chemical features, predicted interaction potential, and 2D structures of Yerba Santa ligands retained among statistically significant apo and/or holo ligand–target comparisons after BH-FDR correction.

Ligand No. and Name	Structural Class	Key Features	Predicted Interactions	2D Structure of Ligand	Statistical Analysis
1 6-methoxynaringenin (5,7,4′-Trihydroxy-6-methoxyflavanone)	Methoxyflavanone	3 OH + OCH_3_	H-bonds + π-π stacking	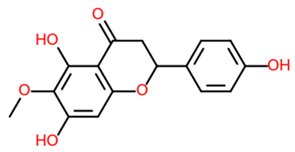	Holo
2 Acacetin (flavone)	Flavone	OH + OCH_3_	H-bonds + π-π stacking	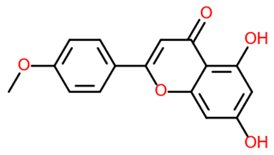	Holo
3 Apigenin (flavone)	Flavone	Planar + 3 OH	H-bonding + π-π stacking	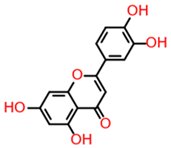	Apo and Holo
4 Astragalin	Flavonol glycoside	Glucose + Flavonol (OH rich)	Extensive H-bonds	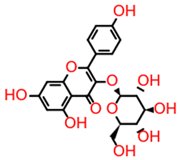	Holo
5 Chrysin (dihydroxy)	Flavone	2 OH; planar	Strong π-π stacking + H-bonding	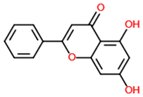	Apo and Holo
6 Cirsimaritin	Methoxyflavone	OH + 2 OCH_3_	H-bonds + hydrophobic fit	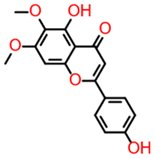	Apo
11 Genkwanin	Methoxyflavone	OH + OCH_3_	π-π stacking	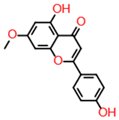	Apo
12 Hesperetin	Flavanone	3OH + OCH_3_	H-bonds + hydrophobic contacts	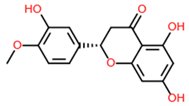	Holo
13 Hispidulin (6-methoxyapigenin)	Methoxyflavone	OH + OCH_3_	π-π stacking + H-bonding	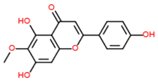	Holo
15 Isoquercetin	Flavonol glycoside	Glucose + Polyphenolic aglycone (OH rich)	Very strong H-bonds	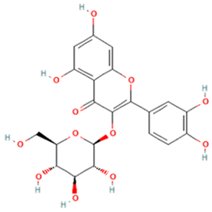	Holo
17 Luteolin	Flavone	4 OH (Polyhydroxylated)	H-bonding + π-π stacking	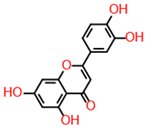	Apo and Holo
19 Naringenin	Flavanone	3 OH	H-bonding + π interactions	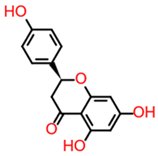	Apo and Holo
20 Pinocembrin	Flavanone	2 OH	π-π stacking + limited H-bonding	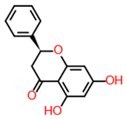	Apo and Holo
21 Rosmarinic acid	Polyphenolic acid	COOH + many OH	H-bonding + potential electrostatic interactions	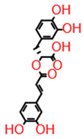	Holo
22 Sakuranetin (flavanone)	Flavanone	OCH_3_ + OH	Hydrophobic contacts + H-bonds	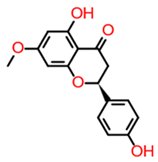	Apo
25a Chrysoeriol (5,7-dihydroxy-2-(4-hydroxy-3-methoxyphenyl)chromen-4-one) (flavone)	Methoxyflavone	OH + OCH_3_	H-bonding + π-π stacking	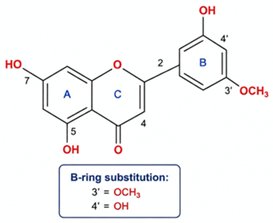	Holo
25b Diosmetin (5,7-dihydroxy-2-(3-hydroxy-4-methoxyphenyl)chromen-4-one))	Methoxyflavone	OH + OCH_3_	Mixed polarity interactions: H-bonding + π–π stacking	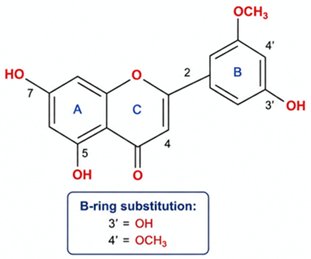	Holo
26a Eupafolin (Nepetin,6-methoxyluteolin)	Methoxyflavone	Multiple OH	H-bonds + π-π stacking	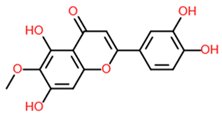	Holo
26b Isorhamnetin	Flavonol	Multiple OH + OCH_3_	H-bonding + hydrophobic interactions	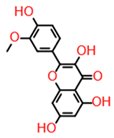	Apo and Holo
27b 6-Methoxysakuranetin	Flavanone (methoxylated)	2 OH + 2 OCH_3_; chiral C-2; non-planar	H-bonding + π-π stacking + hydrophobic interactions	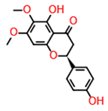	Apo
28b Velutin (flavone)	Flavone	Polyhydroxylated flavone	Strong H-bonding + π-π stacking	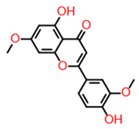	Apo

**Table 5:** Protein Structures (PDB IDs) Associated with Inflammatory Signaling and Alzheimer’s Disease Pathways.

No.	PDB ID	Protein Name (Full, From PDB/UniProt)	Gene Symbol	Protein Name	Structure Type	Properties (Function/Relevance)	Pathway	Reference
1	1E0S	ADP-ribosylation factor 6	ARF6	Arf6	Holo	Vesicle trafficking regulator	Vesicle trafficking	[15]
2	1HZF	Complement component C4, fragment C4d	C4A	C4d fragment	Apo	Complement activation fragment, inflammatory signaling	Complement	[15]
3	1OIW	Ras-related protein Rab-11A	RAB11A	Rab11a	Holo	Vesicle trafficking regulator	Vesicle trafficking	[15]
4	1OWT	Amyloid precursor protein (Cu-binding domain)	APP	APP	Apo	Neurodegeneration-related protein, AD pathology	Amyloid/AD	[24]
5	1T91	Ras-related protein Rab-7A	RAB7A	Rab7s	Holo	Vesicle trafficking regulator	Vesicle trafficking	[25]
6	2A73	Complement component C3	C3	C3	Apo	Central complement protein, inflammatory signaling	Complement	[15]
7	2JG8	Complement component 1q subcomponent subunit B	C1QB	C1q subunit B	Holo	Complement recognition, inflammatory signaling	Complement	[15]
8	2NYY	Complement receptor type 1	CR1	CR1 receptor	Apo	Immune complex clearance, inflammatory signaling	Complement	[15]
9	2ODJ	Delta opioid receptor	OPRD1	δ-opioid receptor	Apo	GPCR, neuroimmune crosstalk	Neuroimmune GPCR	[26]
10	2ODP	Complement component C2, fragment a	C2	C2 fragment a	Apo	Complement protease fragment, inflammatory signaling	Complement	[15]
11	3CU7	Complement component C5	C5	C5	Apo	Complement protein, inflammation & MAC precursor	Complement	[15]
12	3ERB	Complement component C2, fragment b	C2	c2 fragment b	Apo	Complement protease fragment, inflammatory signaling	Complement	[15]
13	7PZC	NLR family pyrin domain-containing 3 (NLRP3)	NLRP3	NLRP3	Holo	Inflammasome sensor, inflammatory signaling	Inflammasome	[27]
14	4BOF	C4b-binding protein alpha chain	C4BPA	C4b-binding protein a chain	Apo	Complement regulator, inflammatory signaling	Complement	[15]
15	4GA7	Plasma protease C1 inhibitor (C1-inhibitor)	SERPING1	C1 inhibitor	Apo	Complement regulator, inflammatory signaling	Complement	[15]
16	4LOT	Complement component 1s	C1S	C1s	Apo	Complement protease, inflammatory signaling	Complement	[15]
17	5JTW	Complement component C4b	C4B	C4b	Apo	Complement fragment, inflammatory signaling	Complement	[15]
18	5O9H	Complement component 5a receptor 1	C5AR1	C5aR1	Holo	GPCR, strong inflammatory signaling	Complement/GPCR	[15]
19	6CM4	Dopamine D2 receptor	DRD2	D2 receptor	Holo	Neurotransmitter GPCR	Neurotransmitter	[28]
20	6CXO	Complement component C9	C9	C9	Apo	MAC component, inflammatory signaling	Complement	[15]
21	6D6U	Gamma-aminobutyric acid receptor subunit alpha	GABRA1	GABA-A receptor α1β2γ2	Holo	Neurotransmitter receptor, inhibitory signaling	Neurotransmitter	[15]
22	6EJ2	Beta-site APP cleaving enzyme 1	BACE1	BACE1 (b-secretase)	Holo	β-secretase, generates Aβ (neurodegeneration-related)	Amyloid/AD	[29]
23	6F39	Complement component 1r	C1R/C1S	C1r-C1s complex	Apo	Complement protease, inflammatory signaling	Complement	[15]
24	6O5E	Vitronectin	VTN	Vitronectin	Apo	Extracellular matrix protein, complement regulation	ECM/complement	[30]
25	7Q6C	Complement component 6	C6	C6	Apo	MAC formation, inflammatory signaling	Complement	[15]
26	7WKI	Complement factor H	CFH	Factor H	Apo	Complement regulator, inflammatory signaling	Complement	[15]
27	7XBW	CX3C chemokine receptor 1	CX3CR1	CX3CR1	Apo	Chemokine receptor, microglia signaling, inflammatory	Microglia signaling	[31]
28	7ZET	Clusterin	CLU	Clusterin	Apo	Complement inhibitor & chaperone, inflammatory regulation	Complement regulation	[15]
29	8HK3	Complement component 3a receptor 1	C3AR1	C3aR1	Apo	GPCR, inflammatory signaling	Complement/GPCR	[15]
30	8WRA	Caspase-1	CASP1	Caspase-1	Apo	Inflammasome effector protease, inflammatory signaling	Inflammasome	[16]

**Table 7:** Functional group annotation and predicted interaction types.

Functional Group	SMILES Pattern	Predicted Interaction Type
Phenolic –OH	OC1=CC=CC=CC=1	H-bond donor and acceptor in interactions with polar residues.
Carboxyl (–COOH)	C(=O)O	Potential H-bond donor and acceptor; the deprotonated carboxylate may form salt bridges and electrostatic interactions with positively charged residues.
Carbonyl (C=O)	C(=O) (in ketones, esters)	H-bond acceptor in ketones, esters, and acids.
Methoxy (–OCH_3_)	OC	H-bond acceptor; may also contribute to hydrophobic contacts and pocket complementarity.
Prenyl group (C_5_)	CC=C(C) or CCC=C(C)	May contribute to hydrophobic and van der Waals contacts within nonpolar pockets.
Glycosyl unit (sugar)	OC(C(O)...)	Multiple H-bond donors and acceptors; increases polarity and may favor interactions in hydrophilic pockets.
Aromatic ring	C1=CC=CC=CC=1	May support π-π stacking with aromatic residues (Phe, Tyr, Trp), and cation-π interactions with Lys and Arg.
Planar flavone core	Fragmented benzopyran w/C2=C3	May support π-π stacking and surface complementarity in flat or open binding sites.
Flavanone C-ring	[C@@H] at C2 (saturated)	Introduces 3D non-planarity that may facilitate adaptation to curved or partially enclosed binding pockets.

## Data Availability

The complete docking dataset, including raw docking scores, ligand structures, protein target preparation parameters, docking pocket coordinates, and MATLAB analysis programs, will be made available following publication of this study. Until then, these materials may be obtained from the corresponding author upon reasonable request.

## Supplementary Materials

The following supporting information can be downloaded at: https://www.mdpi.com/article/10.3390/molecules31203757/s1, Table S1: Structural and chemical information for the remaining Yerba Santa ligands included in the docking library but not meeting the statistical or energetic selection criteria.

## References

[1] Alzheimer’s Association Report (2026). 2026 Alzheimer’s disease facts and figures. Alzheimer’s Dement..

[2] Yin X., Qiu Y., Zhao C., Zhou Z., Bao J., Qian W. (2021). The Role of Amyloid-Beta and Tau in the Early Pathogenesis of Alzheimer’s Disease. Med. Sci. Monit..

[3] Volloch V., Rits-Volloch S. (2023). The Amyloid Cascade Hypothesis 2.0 for Alzheimer’s Disease and Aging-Associated Cognitive Decline: From Molecular Basis to Effective Therapy. Int. J. Mol. Sci..

[4] Passero M., Zhai T., Huang Z. (2023). Investigation of Potential Drug Targets for Cholesterol Regulation to Treat Alzheimer’s Disease. Int. J. Environ. Res. Public Health.

[5] Wang E., Yu K., Cao J., Wang M., Katsel P., Song W.-M., Wang Z., Li Y., Wang X., Wang Q. (2025). Multiscale proteomic modeling reveals protein networks driving Alzheimer’s disease pathogenesis. Cell.

[6] Sharo C., Zhai T., Huang Z. (2024). Investigation of Potential Drug Targets Involved in Inflammation Contributing to Alzheimer’s Disease Progression. Pharmaceuticals.

[7] Wang M., Zhao J., Avula B., Lee J., Upton R., Khan I. (2023). Chemical characterization and quantitative determination of flavonoids and phenolic acids in yerba santa (Eriodictyon spp.) using UHPLC/DAD/Q-ToF. J. Pharm. Biomed. Anal..

[8] Fischer W., Currais A., Liang Z., Pinto A., Maher P. (2019). Old age-associated phenotypic screening for Alzheimer’s disease drug candidates identifies sterubin as a potent neuroprotective compound from Yerba santa. Redox Biol..

[9] Zhai T., Zhang F., Haider S., Kraut D., Huang Z. (2021). An Integrated Computational and Experimental Approach to Identifying Inhibitors for SARS-CoV-2 3CL Protease. Front. Mol. Biosci..

[10] Zhai T., Krass E., Zhang F., Huang Z. (2023). A computational framework for identifying chemical compounds to bind Apolipoprotein E4 for Alzheimer’s disease intervention. Front. Syst. Biol..

[11] Zhang F., Graham J., Zhai T., Liu Y., Huang Z. (2022). Discovery of MurA Inhibitors as Novel Antimicrobials through an Integrated Computational and Experimental Approach. Antibiotics.

[12] Hanes R., Liu Y., Huang Z. (2024). Druggability Analysis of Protein Targets for Drug Discovery to Combat *Listeria monocytogenes*. Microorganisms.

[13] Neves M.A.C., Totrov M., Abagyan R. (2012). Docking and scoring with ICM: The benchmarking results and strategies for improvement. J. Comput. Aided Mol. Des..

[14] Masuda A., Sadato D., Iwadate M. (2024). Improved accuracy of protein-ligand docking prediction using a score distribution learning model. J. Inst. Sci. Eng..

[15] Sharo C., Zhang J., Zhai T., Bao J., Garcia-Epelboim A., Mamourian E., Shen L., Huang Z. (2024). Repurposing FDA-Approved Drugs Against Potential Drug Targets Involved in Brain Inflammation Contributing to Alzheimer’s Disease. Targets.

[16] Heneka M.T., Kummer M.P., Stutz A., Delekate A., Schwartz S., Vieira-Saecker A., Griep A., Axt D., Remus A., Tzeng T.-C. (2013). NLRP3 is activated in Alzheimer’s disease and contributes to pathology in APP/PS1 mice. Nature.

[17] Hong S., Beja-Glasser V.F. (2016). Complement and microglia mediate early synapse loss in Alzheimer mouse models. Science.

[18] Lamers C., Ricklin D., Lambris J.D. (2023). Complement-targeted therapeutics: An emerging field enabled by academic drug discovery. Am. J. Hematol..

[19] Bu J., Zhang Y., Mahan Y., Shi S., Wu X., Zhang X., Wang Z., Zhou L. (2022). Acacetin improves cognitive function of APP/PS1 Alzheimer’s disease model mice via the NLRP3 inflammasome signaling pathway. Transl. Neurosci..

[20] Guo P., Zeng M., Wang S., Cao B., Liu M., Zhang Y., Jia J., Zhang Q., Zhang B., Wang R. (2022). Eriodictyol and Homoeriodictyol Improve Memory Impairment in Aβ25-35-Induced Mice by Inhibiting the NLRP3 Inflammasome. Molecules.

[21] Hochheiser I.V., Pilsl M., Hagelueken G., Moecking J., Marleaux M., Brinkschulte R., Latz E., Engel C., Geyer M. (2022). Structure of the NLRP3 decamer bound to the cytokine release inhibitor CRID3. Nature.

[22] Mursal M., Ahmad M., Hussain S., Khan M.F. (2024). Navigating the Computational Seas: A Comprehensive Overview of Molecular Docking Software in Drug Discovery. Unravelling Molecular Docking—From Theory to Practice.

[23] MolSoft LLC Ligand Docking and Scoring in ICM. https://www.molsoft.com/icmpro/dock-start.html#vls-score.

[24] Long J.M., Holtzman D.M. (2019). Alzheimer Disease: An Update on Pathobiology and Treatment Strategies. Cell.

[25] Guerra F., Bucci C. (2016). Multiple Roles of the Small GTPase Rab7. Cells.

[26] Fenalti G., Giguere P.M., Katritch V., Huang X.P., Thompson A.A., Cherezov V., Roth B.L., Stevens R.C. (2014). Molecular Control of δ-Opioid Receptor Signalling. Nature.

[27] Kelley N., Jeltema D., Duan Y., He Y. (2019). The NLRP3 Inflammasome: An Overview of Mechanisms of Activation and Regulation. Int. J. Mol. Sci..

[28] Beaulieu J.M., Gainetdinov R.R. (2011). The Physiology, Signaling, and Pharmacology of Dopamine Receptors. Pharmacol. Rev..

[29] Chagas Monteiro K.L., dos Santos Alcântara M.G., Lins Freire N.M., Marques Brandão E., Lima do Nascimento V., Maísa dos Santos Viana L.M., Mendonça de Aquino T., Ferreira da Silva-Júnior E. (2023). BACE-1 Inhibitors Targeting Alzheimer’s Disease. Curr. Alzheimer Res..

[30] Ruzha Y., Ni J., Quan Z., Li H., Qing H. (2022). Role of Vitronectin and Its Receptors in Neuronal Function and Neurodegenerative Diseases. Int. J. Mol. Sci..

[31] Lee S., Varvel N.H., Konerth M.E., Xu G., Cardona A.E., Ransohoff R.M., Lamb B.T. (2010). CX3CR1 Deficiency Alters Microglial Activation and Reduces Beta-Amyloid Deposition in Two Alzheimer’s Disease Mouse Models. Am. J. Pathol..

[32] Makabe H., Miyazaki S., Kamo T., Hirota M. (2013). Myrsinoic acid E, an anti-inflammatory compound from Myrsine seguinii. Biosci. Biotechnol. Biochem..

[33] Reichelt K.V., Hartmann B., Weber B., Ley J.P., Krammer G.E., Engel K.H. (2010). Identification of bisprenylated benzoic acid derivatives from Yerba Santa (*Eriodictyon* ssp.) using sensory-guided fractionation. J. Agric. Food Chem..

[34] Lakens D. (2013). Calculating and reporting effect sizes to facilitate cumulative science: A practical primer for t-tests and ANOVAs. Front. Psychol..

[35] Gallivan J.P., Dougherty D.A. (1999). Cation-π interactions in structural biology. Proc. Natl. Acad. Sci. USA.

[36] Almitairi J.O.M., Girija U.V., Furze C.M., Simpson-Gray X., Badakshi F., Marshall J.E., Schwaeble W.J., Mitchell D.A., Moody P.C.E., Wallis R. (2018). Structure of the C1r–C1s interaction of the C1 complex of complement activation. Proc. Natl. Acad. Sci. USA.

