# Supplementary material for: Investigation of Potential Mechanisms of Yerba Santa Compounds Against Alzheimer’s Disease
Source: Molecules. 2026 Oct 19;31(20):3757. doi: 10.3390/molecules31203757 (PMC13707469; doi:10.3390/molecules31203757)
Supplement: Supplementary file 1 [file molecules-31-03757-s001.zip › molecules-4525392-supplementary.pdf]

Table S1. Structural and chemical information for the remaining Yerba Santa ligands included in the docking library but not meeting the statistical or energetic selection criteria is provided in the Supplementary Materials. For completeness, the table includes ligand name, IUPAC designation, molecular formula, molecular weight, and canonical SMILES strings.

Ligand # and Name	Structural Class	Key Features	Predicted Interactions	2D Structure of the Ligand
7<br>Eriodictyol	Flavone	Multiple OH	H-bonding	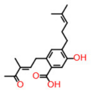
8<br>Eriolic Acid A	Prenylated benzoic acid	COOH + prenyl	Electrostatic + hydrophobic	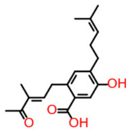
9<br>Eriolic Acid C	Bisrenylated benzoic acid	COOH + 2 prenyls	Salt bridges + deep hydrophobic fit	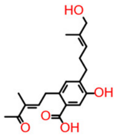
10<br>Eriolic Acid D	Prenylated benzoic acid	COOH + prenyl + OH	Electrostatic + hydrophobic	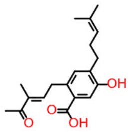
14<br>Homoeriodictyol	Flavanone	OH + OCH3	Moderate H-bonds + hydrophobic interactions	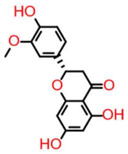
16<br>Jaceosidin	Trihydroxyflavone	Multiple OH + OCH3	Dense H-bond network + π-π stacking	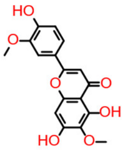
18<br>Melitric acid A (salvianolic acid I) (polylhenolic acid)	Polyphenolic acid	Multiple COOH + many OH	Exceptional H-bonds + salt bridges+ π interactions	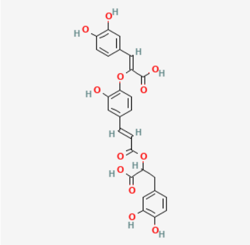
23<br>Salvianolic acid H (3'-O-(8"-Z-Caffeoyl)	Large polyphenolic acid	Multiple COOH + many OH + extended π system	Strongest multi-contact binding (H-bonds, salt bridges, π interactions)	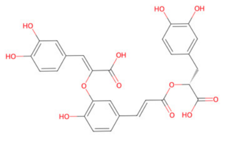

Ligand # and Name	Structural Class	Key Features	Predicted Interactions	2D Structure of the Ligand
24<br>Sterubin (7-O-Methyleriodictyol)	Methoxyflavanone	OH + OCH3 + chiral C-2	H-bonding + π-π stacking + hydrophobic interactions	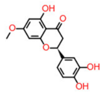
27a<br>5,4'-Dihydroxy-6,7-Dimethoxyflavanone	Dimethoxyflavanone	2 OH + OCH3	π-π stacking + H-bonding	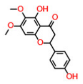
28a<br>Pectolinarigenin (5,7-Dihydroxy-6-methoxy-2-(4-methoxyphenyl)-4-benzopyrone)	Methoxyflavone	Methoxy (OCH3) + phenolic OH	π-π stacking + hydrophobic	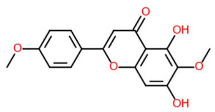
29a<br>Erionic Acid C	Bisprenylated benzoic acid	COOH + 2 prenyl groups	Salt bridges + deep hydrophobic fit	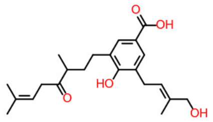
29b<br>Erionic Acid D	Cyclized prenylated benzoic acid	COOH + cyclized prenyl group	Electrostatic + hydrophobic	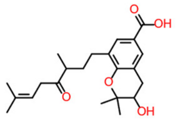
30a<br>Eriolic Acid B	Prenylated benzoic acid	COOH + prenyl	Electrostatic + hydrophobic	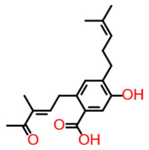
30b<br>Erionic Acid F	Prenylated benzoic acid	COOH + prenyl	Electrostatic + hydrophobic	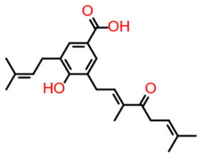
